# Resolving sub-angstrom ambient motion through reconstruction from vibrational spectra

**DOI:** 10.1038/s41467-021-26898-1

**Published:** 2021-11-19

**Authors:** Jack Griffiths, Tamás Földes, Bart de Nijs, Rohit Chikkaraddy, Demelza Wright, William M. Deacon, Dénes Berta, Charlie Readman, David-Benjamin Grys, Edina Rosta, Jeremy J. Baumberg

**Affiliations:** 1 NanoPhotonics Centre, Cavendish Laboratory, University of Cambridge, J J Thomson Avenue, Cambridge, CB3 0HE UK; 2 Department of Chemistry, King’s College London, 7 Trinity Street, London, SE1 1DB UK; 3 Department of Physics and Astronomy, University College London, London, WC1E 6BT UK

**Keywords:** Surface spectroscopy, Organic-inorganic nanostructures, Nanophotonics and plasmonics, Raman spectroscopy

## Abstract

Metal/organic-molecule interactions underpin many key chemistries but occur on sub-nm scales where nanoscale visualisation techniques tend to average over heterogeneous distributions. Single molecule imaging techniques at the atomic scale have found it challenging to track chemical behaviour under ambient conditions. Surface-enhanced Raman spectroscopy can optically monitor the vibrations of single molecules but understanding is limited by the complexity of spectra and mismatch between theory and experiment. We demonstrate that spectra from an optically generated metallic adatom near a molecule of interest can be inverted into dynamic sub-Å metal-molecule interactions using a comprehensive model, revealing anomalous diffusion of a single atom. Transient metal-organic coordination bonds chemically perturb molecular functional groups > 10 bonds away. With continuous improvements in computational methods for modelling large and complex molecular systems, this technique will become increasingly applicable to accurately tracking more complex chemistries.

## Abstract (web summary)

Tracking single molecule movements is a challenging task, but highly desired for applications and fundamental studies. Here the authors reconstruct the sub-angstrom relative movements of a molecule interacting with a metal adatom, by measuring its vibrational spectrum in a self-assembled monolayer, continuously modified by the adatom in a nanoparticle-on-mirror construct.

## Introduction

The push for energy-efficient electronics and growing needs for sustainable manufacturing are driving the miniaturisation of devices and the development of functional nano-components, with promising technologies now incorporating atomic-size features^1–3^. On this same scale, metal-organic interactions underpin many key chemistries from catalysis for feedstocks^4^ to anti-bacterial surfaces^5^ and green initiatives^6^. To extract properties at these atomic length scales, myriad imaging techniques have been developed including electron^7,8^, atomic-force^9^, scanning-tunnelling^10,11^, and tip-enhanced Raman^12,13^ microscopies. Although these are capable of visualising individual atoms, a technique is still required to allow single atom dynamics to be studied at relevant timescales and at ambient conditions. This impedes progress in fields from molecular electronics^14^ to photocatalysis^15^ and electrochemistry^16^. Such a technique is essential to understand how atoms explore their energy landscape and interact with their local chemical environment and to facilitate observations of chemical reactions at their operating temperature.

By placing molecules within the sub-wavelength-confined optical fields of nanostructured plasmonic metals, the enhancement of scattering and absorption opens up new methods of non-destructive and real-time optical interrogation. In nanometre-scale gaps between plasmonic materials, the enhanced field strengths enable optical spectroscopy of fewer than a hundred molecules, in contrast to the trillions observed with classical optics^17^. Single metal adatoms stabilised on the surface of these plasmonic nanocavities further localise the confined optical field to sub-nm^3^ effective volumes termed picocavities^18,19^. This allows single molecules to be optically isolated while the resulting extreme field gradients alter the spectrosopic response, allowing its spectral fingerprint to be distinguished from the ensemble. Such picocavities, which are produced stochastically under laser irradation of the nanocavity, are stable at room temperature depending on nearby chemical species^20^.

**Fig. 1:**
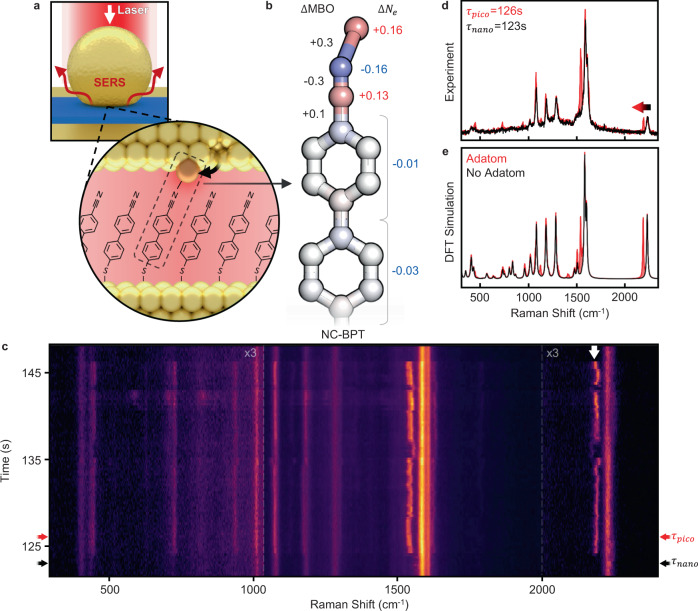
Picocavity formation focuses light onto a single molecule. **a** Nanoparticle-on-mirror (NPoM) structure confines light in nanocavity gap, further confined by single gold adatom (inset) beside molecular monolayer of cyanobiphenyl-4-thiol (NC-BPT). **b** Changes in Mayer bond order (MBO) and charge density (*N*_*e*_) during energetically favourable interaction between Au adatom and C≡N bond. Red indicates an increase in charge density while blue indicates a decrease. **c** SERS spectra of NPoM system over time, including persistent nanocavity lines and transient picocavity lines perturbed by the single adatom (white arrow shows perturbed NC bond). *τ*_nano_ and *τ*_pico_ indicate times before and during the picocavity event shown in **d** single SERS spectra taken before (*τ*_nano_) and after (*τ*_pico_) the formation of a picocavity. **e** DFT spectra of the NC-BPT molecule with and without a gold atom positioned to reproduce experimental spectra, peak height scaled to experiment. Peak ratios without adatom are matched using optical polarisation direction. Peak ratios with adatom omit picocavity enhancement (see text) and thus ad hoc. Weaker C≡N mode in the experiment arises from lower optical collection efficiencies at longer wavelengths.

**Fig. 2:**
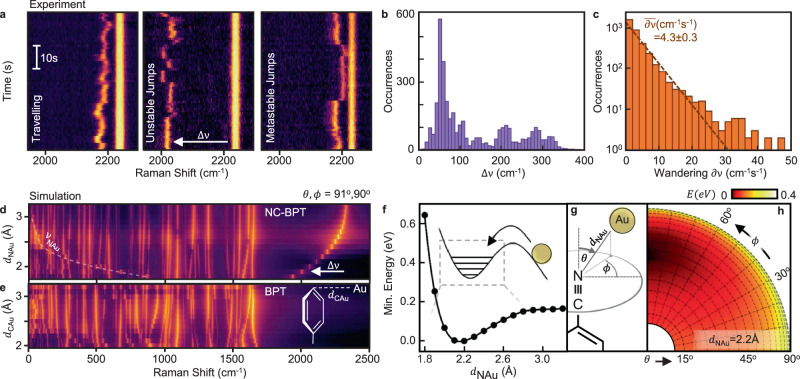
Spectral perturbation varies with adatom position. **a** SERS spectra showing varying dynamical behaviour of the perturbed C≡N vibrational line (Δ*ν*) within the single molecule. **b** Histogram of experimentally observed CN downshifts in NC-BPT from unperturbed vibration energy. **c** Experimentally observed changes in CN vibration energies within 1s, fit to likelihood of wandering $\propto {{\exp }}\left(-\partial \nu /4.3\,{{{{{{\rm{cm}}}}}}}^{-1}\,{{{{{{\rm{s}}}}}}}^{-1}\right)$ (dashed). **d** DFT Raman spectra of NC-BPT *vs d*_NAu_ at fixed *ϕ* = 90°, *θ* = 91° (defined in (**g**), log colour scale). Virtual N−Au vibrational energy indicated by dashed line. **e** DFT Raman spectra of biphenyl-4-thiol (BPT) with adatom approach along energy minimising path (dashed) (log colour scale). **f** System energy *vs d*_NAu_, optimising adatom position angularly. **g** Coordinate system defining adatom position relative to CN bond and upper ring of NC-BPT. **h** Relative system energy with varying adatom angular positions for energetically favourable *d*_NAu_ = 2.2 Å.

Here, the chemical influence of a low-coordination gold adatom on neighbouring organic molecules is probed by the tightly-confined light, revealing their mutual interaction. This is experimentally achieved using surface-enhanced Raman spectroscopy (SERS) to reveal how the vibrational modes of the molecule are continuously modified by the presence of the adatom (Fig. 1). These modified vibrations display both continuous and discrete changes on millisecond timescales (and faster), arising from changes in the relative adatom-molecule position (Figs. 1c, 2a). Developing a comprehensive map of the available parameter space using a density functional theory (DFT)^21^ model allows the experimental spectra to be inverted into sub-Å relative movements of the molecule and adatom. We discuss also the limitations of this approach and highlight new theory needing development.

## Results and discussion

### Experimental nano-construct for picocavity observation

We exploit optical nanocavities based on the robust nanoparticle-on-mirror (NPoM) construct consisting of a gold nanoparticle positioned *d* < 2 nm from a gold mirror (Fig. 1a)^17^. Upon resonant irradiation, the optical coupling between the nanoparticle and its image charges in the mirror leads to strong field confinement in the nanogap in close analogy to a nanoparticle dimer^17^. This mode has an effective volume set by the radius of the nanoparticle (*R* = 40 nm) and the width and refractive index (*n*_*g*_) of the gap as $V\approx R{d}^{2}/{n}_{g}^{2}\, \sim$ 100 nm^3^. The resulting field enhancement offsets low Raman scattering cross-sections allowing for inelastic scattering of light to probe the vibrational modes of any material placed within this volume. Using a self-assembled molecular monolayer (SAM) between the nanoparticle and mirror provides robust Raman signatures due to its close-packed nature^22^. Light-induced picocavities can then interact with these molecules. The cyanobiphenyl-4-thiol (NC-BPT) molecular SAM used here (Fig. 1a) contains a CN headgroup with a distinct high energy SERS line at *ν*_CN_ = 2242 cm^−1^ enabling background-free tracking to study vibrational dynamics. During SAM formation, the molecules bind to a template-stripped gold surface via a thiol linker^23^. When nanoparticles are drop cast onto the sample, >10^7^ separated NPoM constructs are simultaneously created with CN headgroups pointing at the nanoparticle surface. Representative SERS spectra from several million taken on this system (Fig. 1c, d) consistently display the expected strong modes.

During a picocavity event, the strong optical field gradient over a single molecule next to the Au adatom alters the effective Raman selection rules, enabling previously weak modes to become visible and modifying peak ratios. Most obvious is a distinct adatom modified C≡N stretch observed at lower Raman shifts than expected (Fig. 1c, white arrow) in a spectral region where no vibrational peaks are expected to fall. We show these redshifts arise from interactions between the picocavity Au adatom and the nearest C≡N bond, as noted previously^20^. Because only one red-shifted C≡N line is typically seen, this suggests it is from a single molecule^18^, as supported by its narrower linewidth and spectral wandering. Picocavity events in which a modified C≡N stretch is not observed are considered to be atomic features appearing on the gold mirror and are not explored here^20^. We emphasise that such transient SERS peaks from adatom atomic features are pervasive in all nanogap nanocontructs^19,24^.

### Density functional theory of transient adatom binding

DFT calculations show that an adatom in proximity to NC-BPT will energetically favour forming a partial bond with the N (Fig. 1b). This shifts the hybridisation of the nitrogen from *sp* to *sp*^2^, lowering the bond order and vibrational frequency for this individual molecule. Due to conjugation, this perturbation translates to subtle electron density changes across the entire molecule. In the most energetically favourable position, the calculated DFT Mayer bond order (MBO) is 0.3 for the Au-N coordination bond while the C≡N bond order decreases from 3.0 to 2.7. Optimising the position of the gold atom with respect to the molecule allows DFT to accurately reproduce the experimental results (Fig. 1d, e). We find coordination bonds transiently exhibit MBO of up to 0.6 (Supplementary Fig. 1). To highlight more general features in such transient bonding, we also compare below this coordination with that for biphenyl-4-thiol (BPT) without the C≡N headgroup.

To investigate the spectral dynamics more quantitatively, picocavities are repeatedly generated using sporadic 100 ms bursts of 500 μW 633 nm laser irradiation. The short duration favours the creation of a picocavity within a single burst without then destroying it, though their formation is rare (0.08 s^−1^ at this irradiation power) and only ~15% form on the nanoparticle facet^20^. Once formed, they are probed with 25 μW irradiation for 60 consecutive spectra with 1 s integration time. Among trajectories recorded for 87 individual picocavities, the energy dynamics of the perturbed CN vibration displays both continuous and discrete changes on these timescales (Fig. 2a). The downshift of the CN vibration from its unperturbed frequency (Δ*ν*) is observed to range over 400 cm^−1^ but occurs most often around $\Delta \nu$ ~ 50 cm^−1^ (Fig. 2b). As shown below, this compares reasonably with the predicted downshift from DFT of Δ*ν* ≃ 80 cm^−1^ at the optimal N−Au bond-length of *d*_NAu_ = 2.2 Å. The observed changes in downshift between spectra each second, termed here the CN wandering (denoted ∂ν), follow an exponential distribution with an average wandering rate $\bar{\partial \nu } \sim$ (4.3 ± 0.3) cm^−1^ s^−1^ (Fig. 2c). The wanderings show no characteristic frequency dependence, and seem surprisingly slow compared to the sub-ps molecular vibration frequencies, as well as the sub-μs acoustic frequencies of the nanostructure^25^. Similar behaviour is seen in all the systems studied.

We now show that DFT models capture most features of these experimental spectra, which is required to invert them to obtain atomic trajectories. Extending the DFT modelling away from the energetically optimal configuration allows the adatom position relative to the NC-BPT molecule to be tracked by mapping out the spectral response at each configuration (Fig. 2d). Fixing the adatom position relative to the molecule and allowing the rest of the system to geometrically relax, a perturbed vibrational spectrum is simulated. The position of the adatom is tracked using spherical polar coordinates (${d}_{{{{{{\rm{NAu}}}}}}},\theta ,\phi$) with axes defined by the C≡N bond (*z*) and the plane of the upper phenyl ring (*xz* plane) with the N atom as the origin (Fig. 2g). The separation *d*_NAu_ is varied from 1.8–3.2 Å while *θ* and *ϕ* are varied from 0 to 90° (Supplementary Note 1). The minimum energy conformation is found at *d*_NAu_ = 2.2 Å (Fig. 2f), ignoring influences from other SAM molecules or the nearby surface of bulk gold (preliminary inclusion of such effects in calculations is discussed in Supplementary Note 2). At this *d*_NAu_, the system energy is minimised at *ϕ* = 90° (Fig. 2h), reflecting the spatial overlap between the Au valence orbitals and the C≡N *π*-orbital to which they hybridise, and canted at *θ* ~ 40°. The N−Au MBO shows a similar angular dependence maximised at *ϕ* = 90°.

The emergence of this N−Au coordination bond mixes the vibrational modes of the bare molecule, as visualised when plotting the DFT vibrational spectra *vs d*_NAu_ (Fig. 2d). As the adatom approaches, vibrational modes across the spectrum are perturbed with the C≡N vibration clearly lowering in frequency continuously as its bond order drops (Fig. 2d). Changes are also observed in two dominant vibrational modes which stretch the lower and upper phenyl rings at ~1500 cm^−1^. With decreasing *d*_NAu_, the vibrational motion of the top ring is increasingly pinned by the heavy gold adatom. Since the lower ring is already bound to gold, this reduces the molecular asymmetry causing the rings modes to mix (Supplementary Fig. 12). The phenyl-phenyl bond ~1320 cm^−1^ also strengthens, reducing the dihedral angle between the rings (Supplementary Fig. 13) consistent with the previous studies^26^. Further mixing is seen across the Raman spectrum, highlighting electron density reorganisation and consequent changes in bond strengths. A new vibrational pseudo-mode (*ν*_NAu_) rapidly increases in energy as *d*_NAu_ decreases, generating sets of anti-crossings with successively higher energy vibrations (Fig. 2d, dashed line). This directly matches the vibration of the coordination N−Au bond, which increases in strength as the adatom approaches (Supplementary Note 1). This shows that the chemical perturbations extend right across the molecule, including the thiol functional group >10 molecular bonds away from the adatom on the other side of the molecule.

The theoretical energy landscape shows that the metastable Au adatom sits in a well of depth 0.2 eV (Fig. 2f inset), giving an estimated N−Au stretch energy spacing ${\nu }_{{{{{{\rm{NAu}}}}}}} \sim 20$ meV (~162 cm^−1^) as for the N−Au Raman in Fig. 2d. In this anisotropic well, thermal excitations (${k}_{B}T$ = 25 meV) can access only the lowest few states. In a harmonic approximation along the *d*_NAu_ coordinate, a thermal fluctuation of 24 pm is expected, equivalent to changes in *ν*_CN_ of 40 cm^−1^ (Supplementary Note 3), the same order of magnitude as observed for spectral wandering. The softer rotational energy barrier of 0.1 eV along *ϕ* (Fig. 2h) would give torsional oscillations spaced by 3 meV, which are thus more frequently thermally accessed.

To demonstrate the generality of such adatom coordination, we also plot the vibrational modes of biphenyl-4-thiol (BPT) upon adatom perturbation (Fig. 2e). Without the CN headgroup, the adatom approaches the para (top) carbon of the upper phenyl ring along an energetically favourable path perpendicular to the plane of the phenyl ring (*θ,ϕ~*90°). Again, the vibrational modes are strongly modified by the Au adatom as its valence orbitals overlap with the π-orbitals of the ring giving a 0.1 eV local energy well at *d*_CAu_ = 2.2 Å with a MBO of 0.5 for the C−Au bond (Supplementary Fig. 17). Such Au−*π* coordination bonds are likely prevalent in most molecule-surface interactions with picocavities, shifting vibrations by more than thermal energies, and impacting molecular electronics, photocatalysis, and sensing.

It is important to consider whether alternative models can explain the observed spectral dynamics. First, we consider vibrational shifts due to the applied field gradient. As the picocavity field oscillates at optical frequencies, it may induce a vibrational energy change through the AC Stark effect. However, this effect splits vibrational energies symmetrically around the unperturbed centre in contrast to the asymmetric shifts observed. A DC field due to charge accumulation at the adatom position would modify the vibration energy through the DC Stark effect, which for the C≡N functional group scales as (20 ± 5) cm^−1^ V^−1^ ^27,28^. This effect can be replicated in DFT by replacing the adatom with a point charge, but magnitudes as large as 0.5e^−^ change the vibration energy of this bond by only ≤6 cm^−1^ (Supplementary Fig. 18). Similarly, charging the molecule itself fails to recreate the observed perturbations (Supplementary Fig. 19). The ^13^C≡N bond isotopologue would give redshifts of 52 cm^−1^, but is static and cannot explain the observed fluctuating downshifts (Supplementary Fig. 20)^29^.

We thus model the spectral dynamics using a single molecule interacting with an adatom to form coordination bonds, in which the discrete spectral changes observed are explained by the high sensitivity of the perturbed vibrational modes to sub-angstrom relative adatom-molecule motion. We also consider if the adatom might interact consecutively with multiple molecules over the period of observation, but with ~6 Å intermolecular spacing in a bi-phenyl SAM^22^ this would require the adatom to traverse an unfeasibly large distance. Occasionally, the CN-picocavity scans display split C≡N vibrational lines. While not explored in depth here, DFT suggests that these could be caused by the hybridisation of C≡N vibrations from two molecules coupling through mutual interaction with a single adatom (Supplementary Fig. 21). However, the majority of the time (>85%), adatom movement relative to a single molecule describes both jumps and wandering in the perturbed line positions.

### Extracting adatom trajectory on sub-angstrom scale

**Fig. 3:**
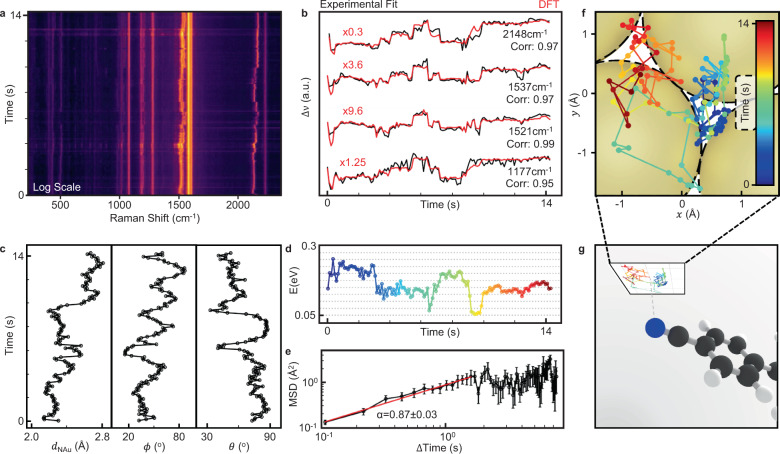
Adatom track to recreate experimental spectra. **a** Experimental picocavity spectra taken with 300 µW 633 nm laser power for 100 ms integration time (log scale). Time starts when picocavity first forms. **b** Extracted picocavity peak positions, overlaid with corresponding peak positions from an optimised adatom trajectory in DFT. These are plotted on individual scales to emphasise the shape of the peak trajectories and the DFT is scaled to overlay (red). High Spearman correlations show good agreement for each peak. **c** Extracted adatom coordinates for this trajectory. **d** DFT system energy along adatom trajectory. Colour refers to time colour bar. **e** Mean square displacement (MSD) of adatom with increasing time window, showing anomalous sub-diffusion on < Å scales. Error bars indicate standard error. **f** Adatom trajectory projected onto a plane, with FCC lattice of underlying nanoparticle facet overlaid and **g** plane shown relative to molecule in 3D. Colours match those in (**f**) indicating time.

As each relative adatom-molecule position generates a unique fingerprint of vibrational lines, picocavity SERS spectra with enough well-resolved peaks should be uniquely invertible to extract a 3D adatom trajectory relative to the NC-BPT molecule. To demonstrate this, a 13 s track with >100 picocavity spectra (Fig. 3a) is taken at higher (300 μW) laser powers to allow shorter 100 ms integration times. Four picocavity peaks are well-enough resolved over all times to compare with the full DFT dataset (linearly interpolating between calculated positions). While the theory captures changes in peak intensity ratios due to the adatom perturbation on the molecular bond orders, it does not fully describe the optical enhancement of the vibrations due to the picocavity field gradient so that peak intensities cannot be used in recovering the adatom position (so far). Fixing the frequency of a single vibrational mode within the DFT collapses the dimensionality of possible adatom positions from the full parameter space to a 2D surface. Fixing additional peaks collapses this further to a 1D line and then a unique point. With uncertainties in experimental vibrational frequencies, confidence in defining the adatom position increases with the number of experimental modes considered (in exact analogy to macroscopic triangulation using GPS satellites). This end goal for the technique requires a very high level of agreement between physical and theoretical mode frequencies. DFT contains intrinsic approximations that typically overestimate vibrational energies. For most applications, this is usually mitigated by applying an empirical scaling factor depending on the level of theory used and the vibrational character of modes^30^, however, some mismatch always remains. For picocavities, the vibrational character of the modes changes dynamically with relative adatom-molecule position. In addition, effects of intermolecular interactions within the SAM are not included (due to rapidly increasing computational complexity). The net result is that no scaling factor for each vibrational mode can place the experimental frequencies within the range of possible frequencies supplied by the DFT for all times (Supplementary Fig. 22). One possible methodology for directly comparing experimental and scaled DFT spectra, in which two separate scaling factors are applied to different regions of the spectrum, is outlined in Supplementary Note 4. With continual improvements in theory and in computational power and resources over time, this simple triangulation of adatom positions will become increasingly practical.

However, an alternative strategy is available based on the correlated changes in vibrational frequencies observed. For this track inversion, an adatom trajectory is instead defined not by matching exact peak positions but instead by matching spectral dynamics. For a given trajectory, the frequencies over time of each peak in theory and experiment can be compared using a Spearman correlation (high Spearman correlation between two sets of values indicates a monotonic relationship). By maximising the averaged Spearman correlation, this forces the trend of each peak perturbation to be captured simultaneously while allowing the correlations to be nonlinear. To test the reproducibility and uniqueness of the solution, five random initial trajectories are maximised in this way, each returning very similar optimised trajectories with averaged correlations ranging from 0.952 to 0.972 (Supplementary Note 5). The similarity of these trajectories is strong evidence for the existence of a globally maximising solution, although small (but non-continuous) alterations may still lead to a small improvement. We show here our maximally-optimised solution with averaged correlation of 0.972 for the four vibrational lines (Fig. 3b, c, Supplementary Movie 1, additional trajectory in Supplementary Fig. 32).

Optimising the average correlation makes this algorithm ambivalent to the relative size of the frequency shifts between theory and experiment. We find the scaling of the perturbations between DFT and experiment varies between vibrational lines. While the shifts in CN peak position are three-fold larger in DFT than experiment, the two phenyl vibrational shifts at ~1500 cm^−1^ are instead 4−10-fold smaller. As expected, this suggests that while our DFT model captures the trend of the peaks the absence of the gold surfaces and surrounding molecules, to which our system would couple through π−π stacking of the phenyl rings, likely has an impact on the scale of these perturbations. This correlation technique requires picocavity peaks to be dynamic over the observed timeframe and for enough to be present to collapse the parameter space to a unique solution. While this limits the inversion of every picocavity track, our algorithm can match crucial information from DFT calculations. Exploring validation from a fifth experimental peak not used for trajectory reconstruction (and not resolvable for all time), shows its predicated dynamics from DFT is well anti-correlated to the extracted experimental trace (Supplementary Fig. 30). This shows that while the comprehensive DFT model captures the character of the experimental perturbations, subtle additional system interactions are still missing but so far are difficult or impractical to include.

Viewing this extracted adatom trajectory in a reference frame with the lower sulphur-bound gold atom fixed at the origin and the plane defined by this Au−S−C junction fixed in orientation, the adatom is found to be restricted close to a flat plane (±0.17 Å) (Supplementary Figs. 33, 34) in which it switches between two distinct clusters. If the movement of the molecule relative to the bulk gold is assumed negligible compared to that of the adatom, this plane can be interpreted as the plane of the nanoparticle facet (Fig. 3f) upon which the trajectory clusters around two sites separated by the 1.7 Å minimum distance between energetically-favourable interstitial sites on a (111) gold surface. This allows the atoms of the nanoparticle facet to be tentatively overlaid over the adatom trajectory (Fig. 3f, dashed), though some molecular motion might also be involved. Among the five optimised trajectories, these sites become increasingly well-defined with the increase in averaged Spearman correlation, converging on the two-site configuration (Supplementary Fig. 28). To probe the robustness of this claim, further trajectories are optimised with artificial noise added to each experimental line from a normal distribution *N*(*μ* *=* 0, σ = 0.5 cm^−1^). While the final Spearman correlation degrades as expected, the two site feature persists (Supplementary Fig. 29). By using this Spearman correlation method, the optimised trajectories are not highly sensitive to changes to the DFT basis set or functional (Supplementary Note 6). The adatom-molecule interaction, as described by the DFT, relaxes slightly over time (Fig. 3d) with transient excitation and relaxation likely arising from transfers with other energy reservoirs in the system such as the interaction between SAM molecules. A discrete drop in energy of 100 meV at 3 s corresponds to when the adatom first leaves the well of its initial interstitial site. This energy is indeed of the order required for an adatom to move away from an interstitial site (Supplementary Note 7). This facet plane defines the molecule as tilted 73° from the vertical, in reasonable agreement with that previously assigned^31^ (Supplementary Note 8). Molecular movement relative to the nanoparticle over relevant timescales can also be considered. Gross nanoparticle motion would modify SERS from all the other molecules in the nanogap (not seen) and from impulsive dynamics is known to damp on <ns timescales with the SAM layer being extremely stiff compared to gold^25^.

Over short timescales where the bounded nature of adatom confinement has little effect, the adatom dynamics show anomalous sub-diffusion (Fig. 3e) with a mean square displacement MSD = Γ∆*t*^*α*^ (over time window ∆*t*) giving *α* = 0.87 ± 0.03 and Γ = (0.92 ± 0.03) Å^2^ s^−1^. This confirms that the sub-atomic thermally-diffusive motion experiences extra hindrance for √MSD < 3 Å (since such sub-diffusion requires additional barriers^32^), which likely comes from the directionality of the bonding involved. This adatom diffusivity agrees in magnitude with previously extracted values for copper adatoms on gold nanocrystals by X-ray diffraction^33^. We point out that using an overdamped Ornstein−Uhlenbeck model, the measured diffusivity $D \sim \Gamma /2\, \sim$ 5 × 10^−21^ m^2^ s^−1^ $\sim {k}_{B}T/(k\tau )$. Given the effective bond spring constant *k* ~ 10 J m^−2^, this suggests that the fluctuation correlation time *τ* ~ 100 ms. The large adatom damping $\gamma =k\tau \sim$ 1 J m^−2^ s thus yields slow dynamics.

This shows how an Au adatom sits in a metastable potential below the facet surface balanced between attractions to the *π* orbitals of the molecule below and the Au facet above (Fig. 2f inset). Many questions still remain about how to consider such atomic motion, which approaches the thermal quantum limit (${k}_{B}T \sim h{\nu }_{{{{{{\rm{NAu}}}}}}}$), and what drives the timescales observed.

### Dynamics with increasing laser power

**Fig. 4:**
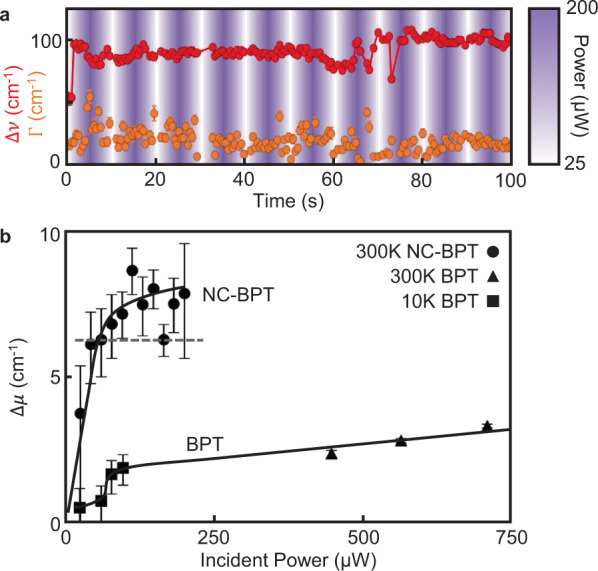
Spectral dynamics power dependence. **a** Full linewidth ($\varGamma$) and downshift $\left(\triangle \nu \right)$ of perturbed NC-BPT CN picocavity peak as incident laser power is cycled between 25 and 200 µW (shading). Neither width nor spectral wandering shows significant power dependence. **b** Effective extent of spectral fluctuations $(\triangle \mu )$ for CN vibration of NC-BPT, BPT picocavity at 10 K, and aggregated picocavities from 106 BPT NPoMs at 300 K (for BPT picocavity lines ~1500 cm^−1^). Error bars indicate standard error. Estimated contribution from 300 K thermal adatom motion along the *d*_NAu_ coordinate for NC-BPT shown as dashed line.

Because picocavities can be detected in real time, the power dependence of their atomic motion can be investigated. During a picocavity event, the laser power is now repeatedly ramped between 25 and 200 µW (Fig. 4a), allowing us to extract how both the linewidth Γ and wandering rate $\bar{\partial \nu }$ of the picocavity lines vary over multiple cycles. Neither is observed to vary significantly, suggesting thermal energies dominate at room temperature. Although each measurement is dependent on the integration time, they can be combined into a single effective width ∆*μ* characterising the spectral dynamics, which is now independent of integration time (Supplementary Note 9). This allows direct comparison between NC-BPT and previous measurements on BPT (Fig. 4b) at both cryogenic^18^ and room temperature^20^ (on hundreds of NPoMs). For BPT, picocavity lines are chosen that appear near 1500 cm^−1^. To compare these to DFT, *d*_NAu_ is scanned to reveal the energy required to shift the CN vibration frequency (Fig. 2d, e), thus resolving thermal contributions at 300 K of $\triangle {\mu }_{{{{{{\rm{NC}}}}}}-{{{{{\rm{BPT}}}}}}} \sim \,$ 6 cm^−1^, in good agreement with observations (note this assumes a simple approximation of the full 3D energy landscape, Supplementary Note 9). Similar estimates for BPT give $\triangle {\mu }_{{{{{{\rm{BPT}}}}}}} \sim$ 10 cm^−1^, overestimating the observed value by threefold and suggesting additional factors at play. This is further evidence for the importance of intermolecular interactions that can modify the scale of phenyl ring perturbations in this spectral region. Experimentally, only at low temperatures is there a clear contribution from the laser vibrational heating of the molecule in picocavities.

In conclusion, we demonstrate how single-molecule spectroscopy can be combined with comprehensive DFT modelling to visualise metal−molecule interactions in ambient conditions. The reconstruction of individual atomic trajectories for all atoms involved reveals the sub-angstrom relative anomalous diffusion of individual gold adatoms as they thermally sample the local energy landscape and thus has great potential to provide powerful insights for heterogeneous catalysis. This opens the way to systematic exploration, for instance of thermal activation in these surface potentials and its competition with optical energy to control the wandering observed, as well as how these potentials are sculpted by different molecular moieties. With single picocavities optically induced here on initially flat facets, roughness plays no role, but catalytic sites such as steps can also now be explored. This provides a valuable tool to study the behaviour of single atoms, which will lead to invaluable insights in widespread fields from nanoscale crystallinity and metal−protein interactions to molecular electronics. The generality of this technique is currently limited by the requirement for dynamic vibrational modes that constrain the trajectories, and by models which capture more complex interactions within the experimental system. However the continued development of DFT through advanced codes and increased computational resources, which allow for larger close-packed systems and complex inter-molecule interactions to be modelled, gives optimism for continual improvement in this method.

## Methods

### Sample preparation

Gold nanoparticles were purchased from BBI Solutions (80 nm citrate capped) and stored at 5 °C. All chemicals were purchased from Sigma Aldrich and used as received. Template-stripped gold was prepared by cleaning a silicon wafer with Decon 90 and rinsing twice with water, ethanol, and isopropyl alcohol, and blown dry using nitrogen. A 100 nm thick layer of gold was deposited at a rate of 1 Å∙s^−1^ using an electron-beam evaporator. The wafer was heated to 60 °C and ~0.5 cm^2^ silicon wafer pieces were glued onto the gold side using ~ µL of Epo-Tek 377 epoxy glue. The wafer was heated to 150 °C and left to cure the epoxy overnight, after which it was gradually cooled to room temperature at a rate of $\sim$1 °C∙min^−1^ to prevent crack formation. When required, a piece of glued silicon was peeled off exposing a clean flat gold surface transferred from the wafer. This was immediately submerged in a 1 mM solution of cyanobiphenyl-4-thiol (NC-BPT, 97%, Sigma Aldrich) in ethanol (200 proof, anhydrous, ≥99.5%) for 18 h. The sample was then rinsed with excess of ethanol and blown dry using nitrogen.

For nanoparticle deposition, 100 µL of colloidal gold suspension (80 nm, citrate capped, BBI solution, OD1) was mixed with 5 µL of a 20 mM solution of KCl and allowed to mix for 30 s. A 30 µL droplet was deposited on the SAM functionalised gold substrate and left for 30 s, after which the droplet was rinsed off with an excess of deionised water and blown dry using nitrogen.

### SERS measurements

NPoM constructs were imaged using an Olympus BX51 microscope in dark field configuration using a 100x Zeiss Ex Epiplan-Neofluar 0.9NA objective lens and a Lumenera Infinity 2 CCD. A Prior Scientific motorised stage was used to centre nanoparticles. A 632.8 nm Integrated Optics SLM VBG diode laser focussed to a diffraction-limited spot was used to excite Raman scattering, with scattered light imaged onto an Andor Newton 970 EMCCD through a HORIBA Triax 320 spectrometer using a 600 l/mm diffraction grating with 750 nm blaze. The elastically scattered light was removed using two Thorlabs 633 nm notch filters and laser power on the sample was measured using a Thorlabs PM16-121 power metre.

### Density functional theory

All DFT calculations were carried out with Gaussian09 Revision E programme suite^21^. In these computations, the B3LYP^34^ hybrid density functional, and D3 dispersion correction with Becke−Johnson damping^35^ were employed with the Def2TZVP basis set^36,37^. The adatom was modelled as a single gold atom, while the gold mirror was modelled as one or two gold atoms attached to the sulfur atom. The number of gold atoms was chosen for each case to retain the singlet electronic state of the system. Unrestricted self-consistent field calculations were performed to ensure electronic stability for the wavefunction. For more details see Supplementary Notes 1, 2.

## Supplementary information

Supplementary Information

Description of Additional Supplementary Files

Supplementary Movie 1

## Data Availability

The data presented in this study is available within the Apollo repository at the following 10.17863/CAM.76655.
