# Supplementary material for: Resolving sub-angstrom ambient motion through reconstruction from vibrational spectra
Source: Nat Commun. 2021 Nov 19;12:6759. doi: 10.1038/s41467-021-26898-1 (PMC8604935; doi:10.1038/s41467-021-26898-1)
Supplement: Supplementary file 1 — Supplementary Information [file 41467_2021_26898_MOESM1_ESM.pdf]

Resolving Sub-Angstrom Ambient Motion through Reconstructions from Vibrational Spectroscopy

Supplementary Information

Jack Griffiths,[1] Tamás Földes,[2,3] Bart de Nijs,[1,*] Rohit Chikkaraddy,[1] Demelza Wright,[1] William M. Deacon,[1] Dénes Berta,[2,3] Charlie Readman,[1] David-Benjamin Grys,[1] Edina Rosta,[2,3] Jeremy J. Baumberg[1,*]

[1] NanoPhotonics Centre, Cavendish Laboratory, J J Thomson Avenue, University of Cambridge, CB3 0HE, UK

[2] Department of Chemistry, King's College London, 7 Trinity Street, London SE1 1DB, United Kingdom

[3] Department of Physics and Astronomy, University College London, London WC1E 6BT, United Kingdom

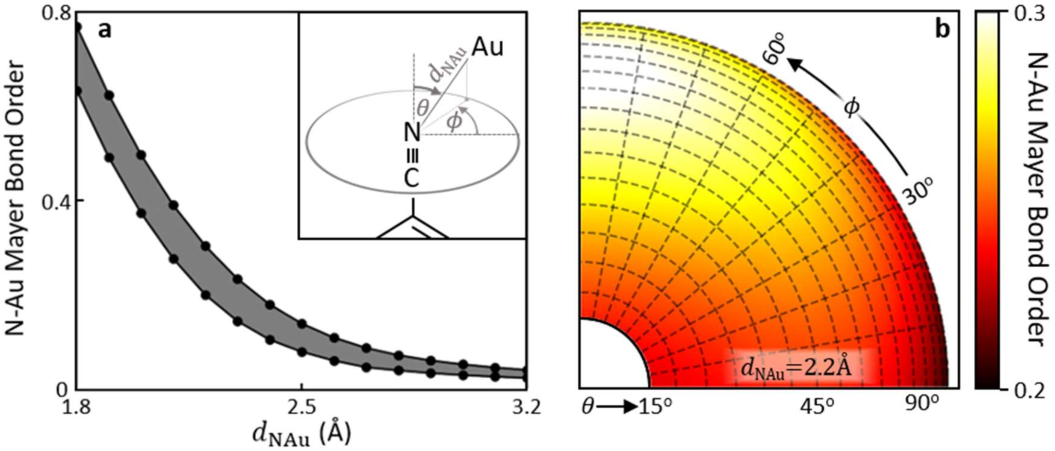

Supplementary Figure 1 | Adatom-Nitrogen bond order. a, The range of Mayer bond orders possible between the N atom in NC-BPT and the Au adatom separated from it by d_{NAu}, varying with the angular position of the adatom relative to the molecule. **b,** the Mayer bond order with varying angular position at a fixed $d_{\mathrm{NAu}} = 2.2$Å. Coordinate system shown in inset.

Supplementary Note 1 | DFT calculations

We carried out density functional theory (DFT) with the Gaussian09RevE program suite for NC-BPT analogues (Supplementary Figure 2). To account for the bulk gold in the experiments, we modelled the system by adding a few interacting gold atoms connected to the thiol sulfur atom, or closely located near the CN nitrogen. The number of gold atoms were chosen for every case to keep an even number of electrons in the system. For this reason, while the NC-BPT-Au system has a single gold atom bonded to the S atom, the Au⋯NC-BPT-Au-Au includes two additional gold atoms – one on each side of the molecule. We used the B3LYP hybrid exchange correlation functional with Grimme's D3 dispersion correction with Becke-Johnson damping (B3LYP-D3BJ). To ensure the accuracy of our calculations we employed ultrafine grids for all calculations, and several tests were carried out for the spin state considering singlet restricted, singlet unrestricted and triplet unrestricted states (Supplementary Table 1 and Supplementary Figure 3) and Def2-SVP, Def2-SVPD and Def2-TZVP basis

sets (Supplementary Figure 4). Based on our results we chose the Def2-TZVP triple-ζ basis set and carried out all calculations with unrestricted wave functions from a desymmetrised guess. We used vibrational analysis to obtain polarizability derivatives and to calculate the Raman intensity spectra.

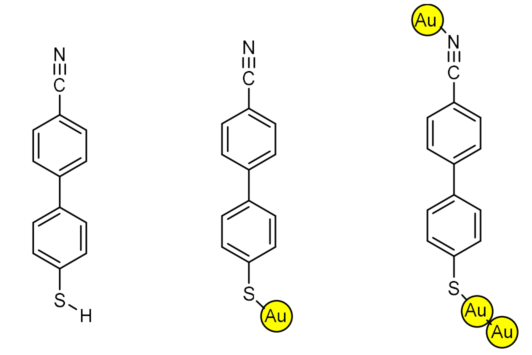

NC-BPT-Au **NC-BPT-Au** **Au···NC-BPT-Au-Au**

Supplementary Figure 2 | Molecular species investigated via DFT calculations. Molecular structures for three versions of the NC-BPT molecule interacting with differing numbers of gold atoms. An odd number of gold atoms is maintained in order to retain an even number of electrons overall.

Supplementary Table 1 | Electronic energies and spins obtained for the Au···NC-BPT-Au-Au complex with different spin states.

Wave function	Spin state	<S^2>	Electronic energy (Hartree)
Restricted	Singlet	0.0000	-1360.051346
Unrestricted	Singlet	0.8021	-1360.060312
Unrestricted	Triplet	2.0118	-1360.056886

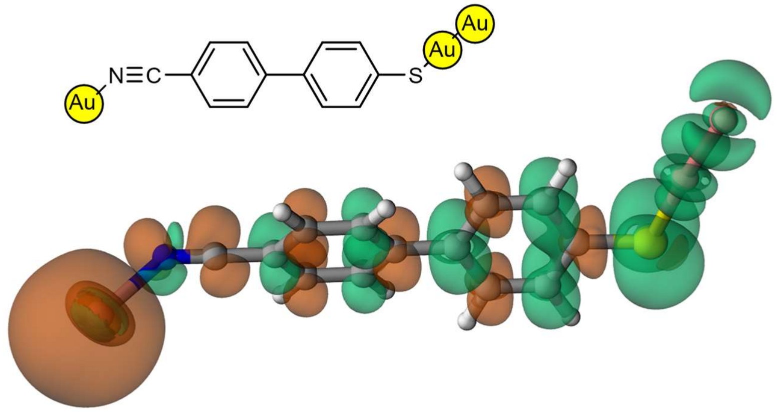

Supplementary Figure 3 | Spin density obtained for Au···NC-BPT-Au-Au with open-shell singlet electronic configuration.

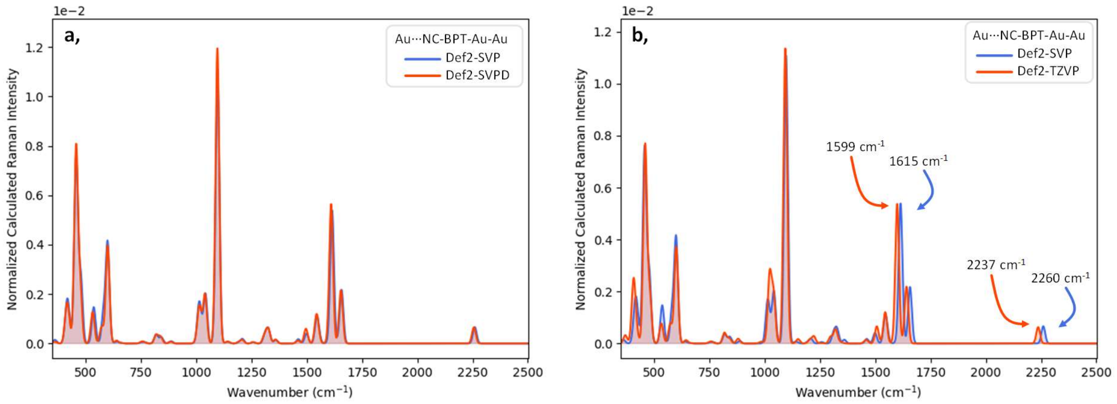

Supplementary Figure 4 | Overlay spectra to compare the performance of different basis sets. a, Comparison between Def2-SVP and Def2-SVPD basis sets shows no significant change in the Raman spectrum with the addition of diffuse functions to the basis set. **b**, Comparison between Def2-SVP and Def2-TZVP basis sets reveals significant differences at larger wavenumbers.

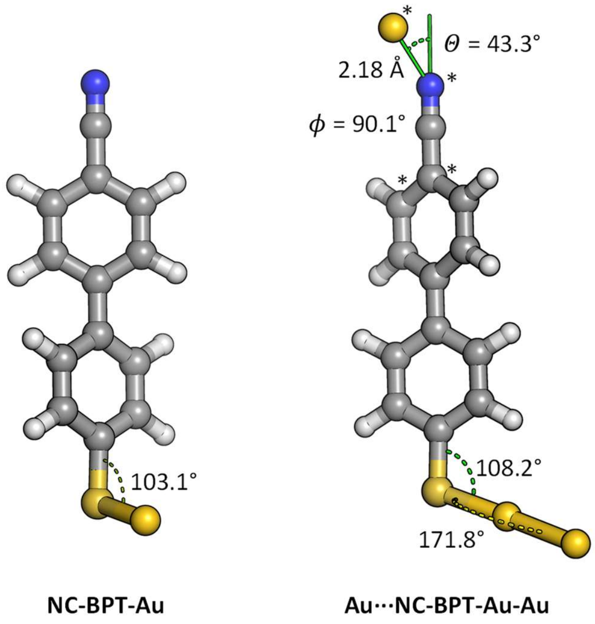

Supplementary Figure 5 | Optimised structures of NC-BPT-Au and Au···NC BPT-Au-Au. These geometries are obtained from free minimizations without any constraints applied on the atoms.

Adatom tracking

Gold adatom positions were generated systematically around the N atom in the Au···NC-BPT-Au-Au by varying three interatomic coordinates:

1. The distance between the Au and N atoms was increased from 1.8 Å to 3.2 Å in steps of 0.1 Å.
2. The Au-N-C angle (which is the complementary angle to Θ in Supplementary Figure 5) was scanned from 89° to 169° with a step size of 10°. Thus the polar coordinate Θ was scanned from 91° to 11°.
3. The torsion of the N-Au axis with respect to the plane of the upper phenyl ring (polar coordinate ϕ) was scanned as the dihedral angle defined by the atoms Au-N-C-C changes, where the two

carbon atoms are the ipso and ortho carbon atoms of the upper phenyl ring, respectively. In order to increase the efficiency of our calculations and lower the computational costs, we operated with the assumption that the results are symmetrical across the two symmetry planes of the phenyl ring. Accordingly, we carried out the scan along this third coordinate between 0° and 90° with a stepsize of 10°, and generated the the datasets for the three remaining quadrants by reflecting the obtained data (Supplementary Figure 6).

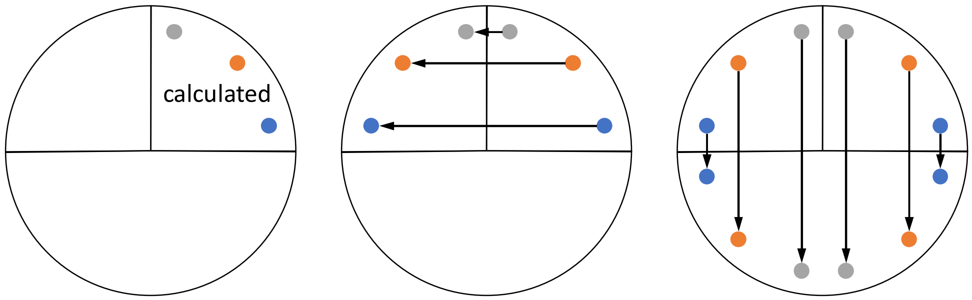

Supplementary Figure 6 | Schematic representation of expanding the obtained data over all four quadrants.

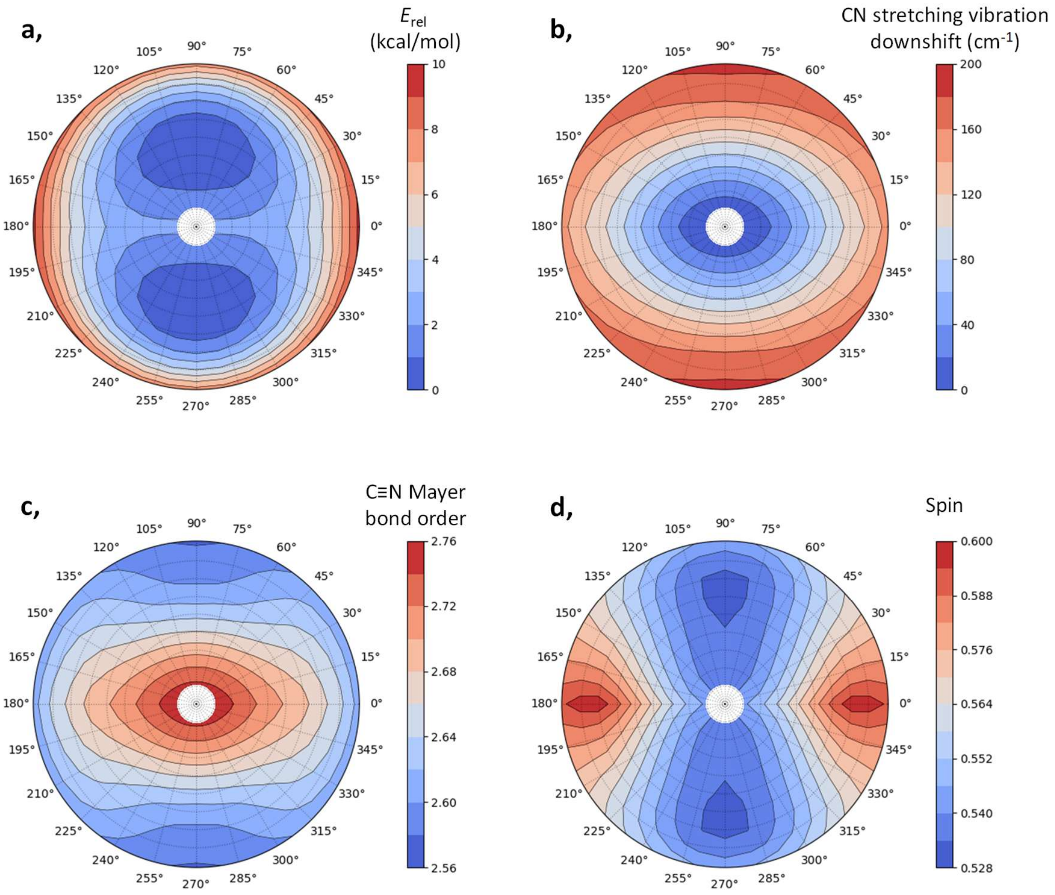

Supplementary Figure 7 | Positional dependence of parameters obtained from adatom tracking with fixed N-Au distance of 2.2Å. a, Relative electronic energy in kcal/mol units. **b,** Downshift of the CN stretching vibrational mode in cm^{-1} units. **c,** Mayer bond order obtained for the C≡N bond. **d,** Total spin of the system.

Au-N vibration analysis

The vibration analysis for the non-equilibrium configurations in the three-dimensional potential energy scan shows significant perturbation in the low frequency part of the spectrum (Supplementary Figure 8a). The extent of the affected region depends on the corresponding N-Au distance. This effect arises from the mixing of a new distance-dependent mode obtained from vibrational analysis. Here we provide a quantitative model which explains this behaviour. For this analysis spherical parameters ϕ=90°, θ=91° were chosen in agreement with Figure 2d of the manuscript. The relative electronic energy of the system as a function of N-Au distance is depicted in Supplementary Figure 8b. A 7th order polynomial is fitted to the discrete datapoints and used for further analysis.

In vibrational analysis, the vibrational frequency (ν_i) corresponding to a vibrational mode with force constant D_i and reduced mass μ_i can be calculated as:

$$\nu_i = \frac{1}{2\pi}\sqrt{\frac{D_i}{\mu_i}}$$

In this formula, the force constant D_i is the second derivative of potential energy along the vibrational coordinate. The second derivative of the fitted polynomial (in Supplementary Figure 8c) is thus used to approximate the vibrational frequency of the related vibrational mode.

To estimate the corresponding reduced mass, one vibrational mode was selected for each of the two cases with N-Au distances of 1.8 Å (Supplementary Figure 8e) and 3.0 Å (Supplementary Figure 8f). In these modes, the stretching motion of the N-Au atom pair is heavily involved. These clearly demonstrate that at 1.8 Å distance the vibrational motion mainly concerns the interacting N and Au atoms, while the vibration extends to additional atoms of the NC-BPT molecule at larger N-Au distances.

In our approach, we approximate the intramolecular motion as a linear translation of the Au atom and a rotation of the involved atoms of the NC-BPT around an origin. At 1.8 Å only the N atom moves, which is modelled as a rotation around the C atom of the N≡C group. Meanwhile at 3.0 Å, all atoms belonging to the upper phenyl ring and the N≡C group seem to rotate around the ipso-C atom of the lower phenyl ring.

The forces acting on the interacting parties are equal in magnitude and opposite in sign:

$$F_{\mathrm{Au}} = -F_{\mathrm{NC-BPT}}$$

$$F_{\mathrm{Au}} = m_{\mathrm{Au}} \cdot a_{\mathrm{Au}}$$

$$-F_{\mathrm{NC-BPT}} = -I_{\mathrm{NC-BPT}} \cdot \ddot{\varphi}_{\mathrm{NC-BPT}} = -I_{\mathrm{NC-}} \quad \cdot \frac{a_{\mathrm{N}}}{r_{\mathrm{N}}}$$

where m_{Au} is the mass of the Au atom (196.97 atomics units a.u.), $I_{\mathrm{NC-BPT}}$ is the moment of inertia calculated for the moving part of the NC-BPT, r_{N} is the distance of the N atom from the centre of rotation, $\ddot{\varphi}_{\mathrm{NC-BPT}}$ is the angular acceleration of the rotating part of the NC-BPT, and $a_{\mathrm{Au,N}}$ are the linear accelerations of the Au and N atoms respectively. From these a_{N} can be expressed as:

$$a_{\mathrm{N}} = -a_{\mathrm{Au}} \cdot \frac{m_{\mathrm{Au}} \cdot r_{\mathrm{N}}}{I_{\mathrm{NC-BP}}}$$

The relative acceleration of the N and Au atoms (a_{rel}) is:

$$a_{\mathrm{rel}} = a_{\mathrm{Au}} - a_{\mathrm{N}} = a_{\mathrm{Au}} + a_{\mathrm{Au}} \cdot \frac{m_{\mathrm{Au}} \cdot r_{\mathrm{N}}}{I_{\mathrm{NC-BPT}}} = m_{\mathrm{Au}} \cdot a_{\mathrm{Au}} \cdot \frac{I_{\mathrm{NC-BPT}} + m_{\mathrm{Au}} \cdot r_{\mathrm{N}}}{I_{\mathrm{NC-BPT}} \cdot m_{\mathrm{Au}}}$$

$$= F_{\mathrm{Au}} \cdot \frac{I_{\mathrm{NC-BPT}} + m_{\mathrm{Au}} \cdot r_{\mathrm{N}}}{I_{\mathrm{NC-B}} \quad \cdot m_{\mathrm{Au}}}$$

The second component is the reduced mass of the system for the corresponding vibrational mode involving the N-Au stretching motion,

$$\mu = \frac{I_{\mathrm{NC-B}} \quad \cdot m_{\mathrm{Au}}}{I_{\mathrm{NC-B}} \quad + m_{\mathrm{Au}} \cdot r_{\mathrm{N}}}$$

As the vibrational motion gradually shifts from a single N atom to the N≡C group and the upper phenyl ring of the NC-BPT, we decided to calculate the vibrational frequencies with both reduced masses as extrema for the system. For single adatom movement the reduced mass is 14.92 a.u., which gives the green points in Supplementary Figure 8d. At large frequencies (small N-Au distances) the obtained curve lies close to the line of perturbations across the spectra (white curve). At larger distances however, the approximation gives larger vibrational frequencies than the expected values. This supports the approximation that at lower N-Au distances the emerging vibration mainly concerns the N atom of the NC-BPT molecule. At longer distances (>3 Å) deviations are caused by poorer polynomial fitting at the edges of the dataset (indicated with open markers). For the second case the obtained reduced mass is 111.38 a.u., which gives lower vibration frequencies for the same second derivatives (blue points in Supplementary Figure 8d). This latter series of data is indeed closer to the observed values at larger N-Au distances.

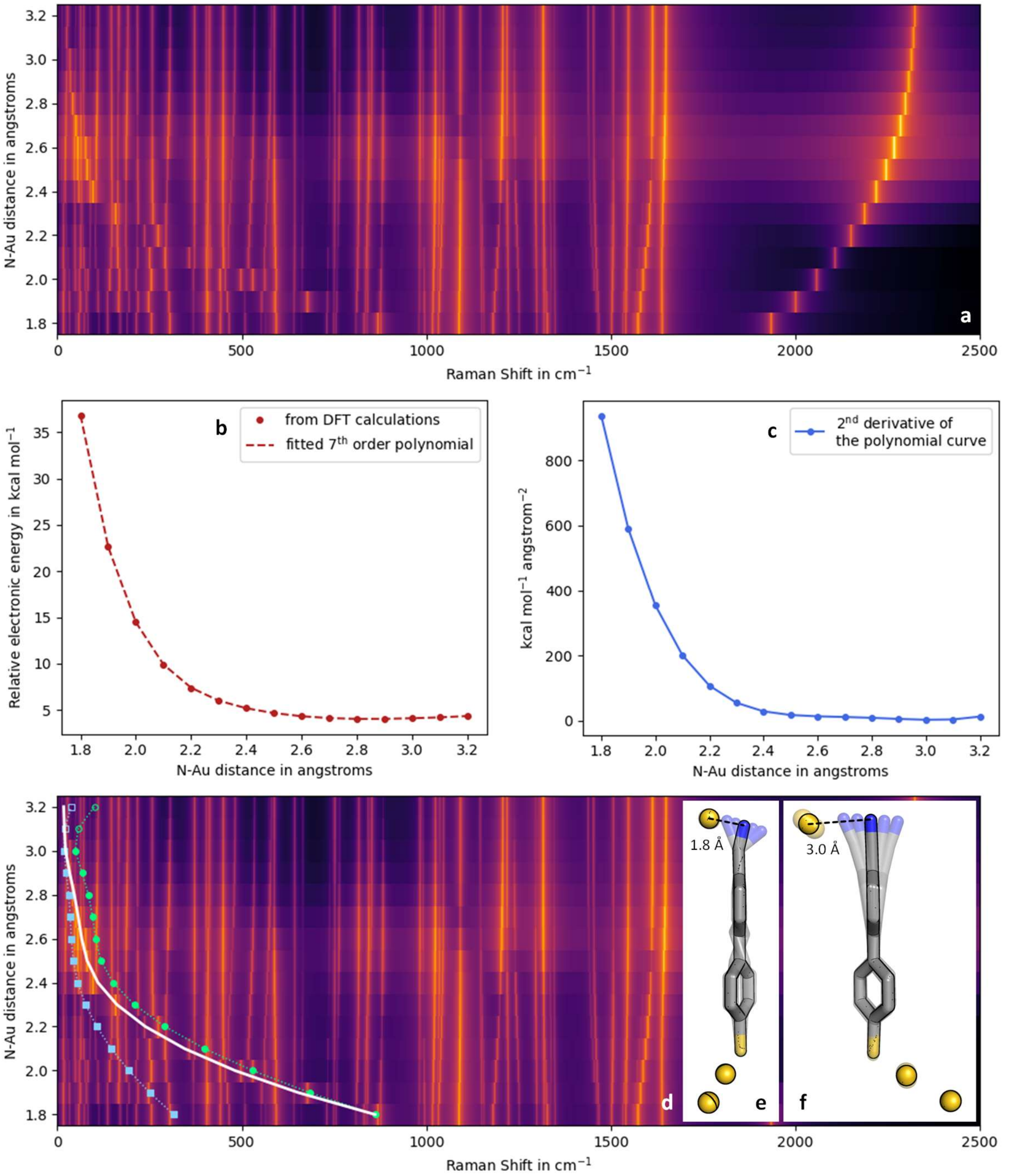

Supplementary Figure 8 | Influence of the fixed N-Au distance on the Raman spectra. a, Raman spectra obtained for adatom tracking with spherical parameters ϕ=90º, θ=91º **b,** Relative electronic energy of each chosen geometry. **c,** Second derivative of the fitted 7th order polynomial. **d,** Position of the perturbation in the Raman spectra (white continuous line), estimated position obtained for N atom movement (green dotted curve), estimated position obtained for the combined movement of the N≡C and the upper phenyl groups (blue dotted curve). Points at 3.1 Å and 3.2 Å obtained with higher uncertainty (empty markers). **e,** Atomic displacement for N-Au stretching vibration at 1.8 Å N-Au distance. **f,** Atomic displacement for N-Au stretching vibration at 3.0 Å N-Au distance.

Supplementary Note 2 |Models of Bulk Gold and Intermolecular Interactions

The Raman spectra of gold-attached NC-BPT molecules are calculated (Supplementary Figure 9) and the mirror surface is modelled with one (**a**), five (**b**) and thirteen (**c**) gold atoms. The obtained spectra of (**b**) and (**c**) are highly similar to that of (**a**)**.** The root-mean-square deviation (RMSD) of peak positions in the range 700-2500 cm^{-1} compared to (**a**) is 2.9 cm^{-1} for (**b**) and 2.1 cm^{-1} for (**c**). Moreover, all three spectra show high similarity to that experimentally obtained in Figure 1 of the manuscript. We note that while the Raman spectra are similar, the required computational time is more than five times longer for the largest system (**c**) than for the smallest (**a**).

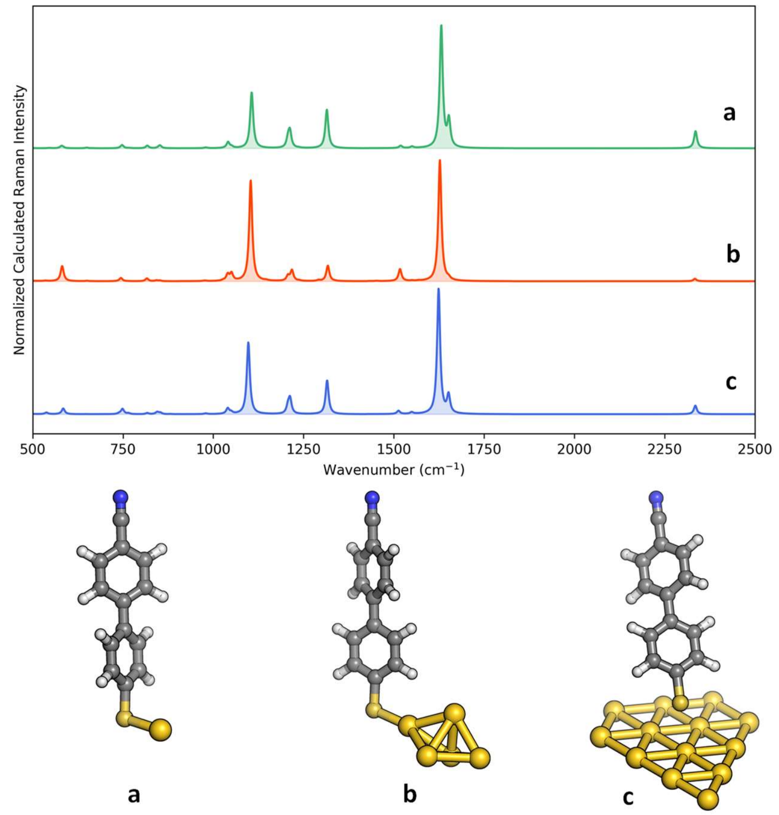

Supplementary Figure 9 | Thiol-Gold binding in the presence of a CN adatom. Raman spectra calculated for the adatom-complexed NC-BPT molecule attached to one (**a**), five (**b**), and thirteen (**c**) gold atoms via the thiol group.

The same comparison for the adatom-complexed NC-BPT molecule would require a reference spectrum for a well-defined structure, to which one could compare all corresponding computationally obtained spectra. Due to the nature of the investigated system, no such reference can be identified. Our calculations for two, four, and twelve gold atoms used as models for the gold mirror produce spectra that are less similar at first glance (Supplementary Figure 10). However, detailed comparison reveals that the peak positions are still similar, and that differences in peak intensities cause most of the apparent dissimilarity. The absolute RMSD of peak positions in the range 700-2500 cm^{-1} compared to (**d**) is 6.8 cm^{-1} for (**e**), and 5.8 cm^{-1} for (**f**). This is still smaller than the absolute RMSD of 12.4 cm^{-1} for (**d**) compared to (**a**).

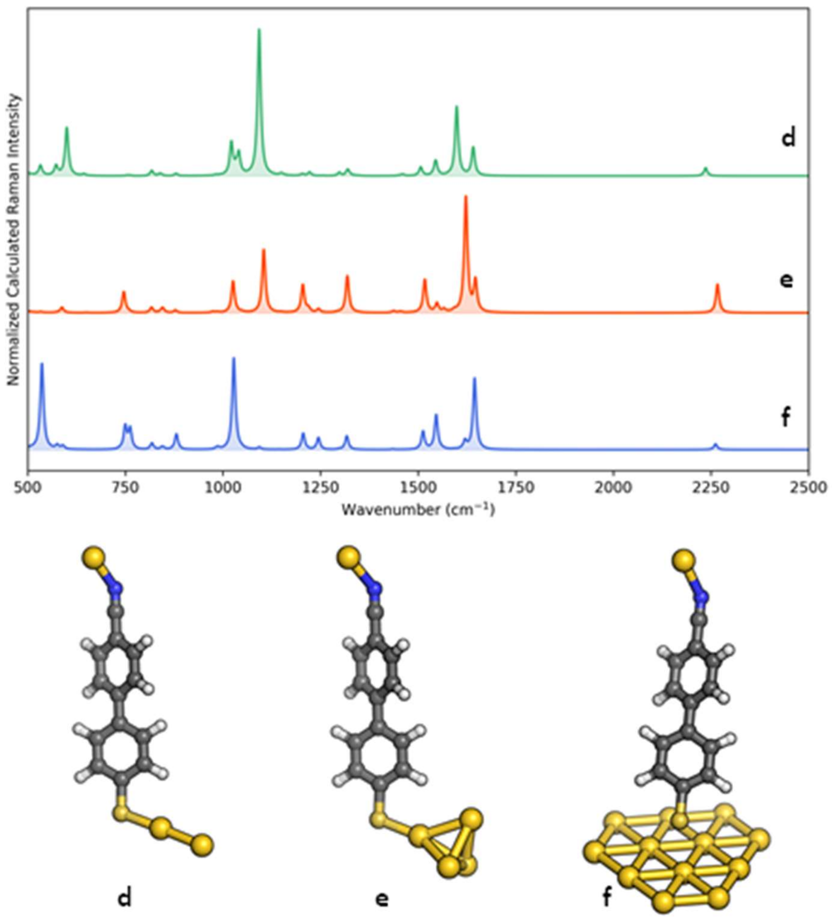

Supplementary Figure 10 | Thiol-Gold binding in the presence of a CN adatom. Raman spectra calculated for the adatom-complexed NC-BPT molecule attached to two (**d**), four (**e**), and twelve (**f**) gold atoms via the thiol group.

In addition, we tested the interaction between the nitrile of the NC-BPT and a gold slab comprised of 12 gold atoms (Supplementary Figure 11). The obtained Raman spectrum shows high similarity to the one obtained for the non-interacting, gold-attached NC-BPT molecule (**a** vs. **c**), unlike the one interacting with a single gold atom (**b**). This alludes to the importance of the gold coordination in the interaction with the nitrile N atom, which results in practically no change in peak positions for the gold slab with large coordination number. In contrast, the gold adatom with low coordination (modelled with a single gold atom) changes the peak positions significantly. This is also reflected in the atomic separations, with the N-slab distance (2.81 Å) much larger than the N-Au distance (2.16 Å).

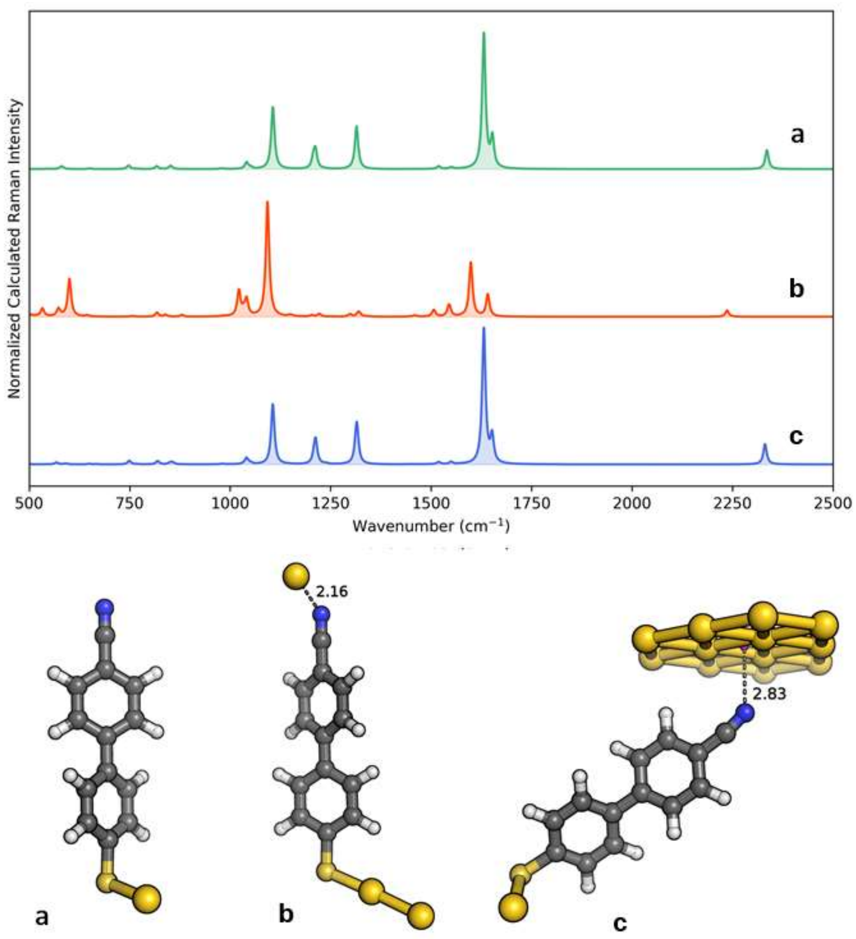

Supplementary Figure 11 | Adatom versus gold slab interaction. Raman spectra calculated for the gold-attached NC-BPT molecule (**a**) without adatom interaction, (**b**) with the adatom modelled as a single gold atom, and (**c**) modelling the gold nanoparticle surface with a slab comprised of 12 gold atoms.

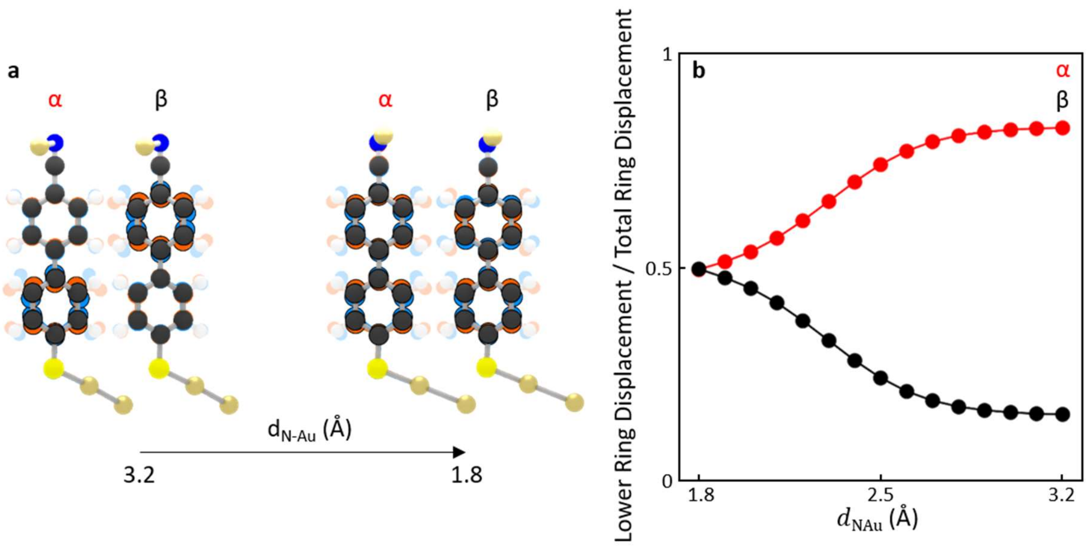

Supplementary Figure 12 | Ring vibrational modes mixing with adatom interaction. a, Schematic of the two strong phenyl ring vibrational modes as the adatom approaches perpendicular to the upper ring, from DFT. At large distance, these vibrations are localised on either ring. They mix into symmetric and antisymmetric modes delocalised across both rings as the adatom approaches. **b,** Fraction of total ring carbon atom movement associated with each vibration contained in the lower ring, showing delocalisation as the adatom approaches

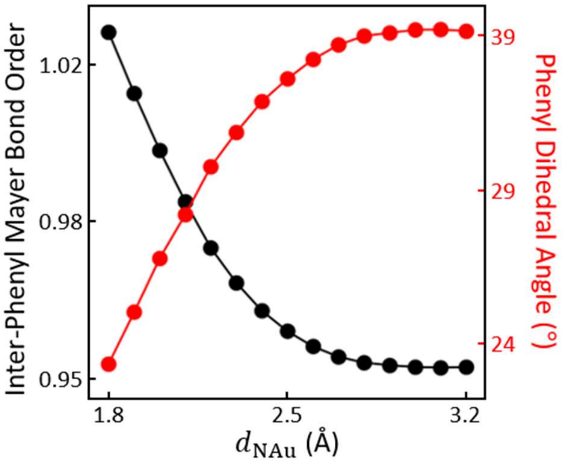

Supplementary Figure 13 | Phenyl Ring Dihedral Angle with Adatom Interaction. C-C Mayer bond order between the phenyl rings of NC-BPT increasing as the Au adatom approaches the N perpendicular to the upper phenyl ring. The dihedral angle between the phenyl rings simultaneously decreases.

Supplementary Note 3 | Quantum Harmonic Oscillator

We model the energy landscape of the adatom interacting with the NC-BPT molecule as harmonic along the d_{NAu} coordinate passing through the minimum energy configuration. Around the energetically favourable position, this energy potential has an effective spring constant of $k = 2.84$ eVÅ^{-2}. We take the mass (m) of the Au-N system as 14.92 atomic mass units (see above).

The energy level spacing of a quantum harmonic oscillator (QHO) is given by

$$\hbar\omega = \hbar\sqrt{\frac{k}{m}} = 20\ \mathrm{meV}$$

whilst the zero-point motion in the ground state is given by

$$\langle x^2\rangle^{\frac{1}{2}} = \sqrt{\frac{\hbar}{2m\omega}} = 16\ \mathrm{pm}\,.$$

Taking a thermal energy of $k_B T$=25 meV, this QHO exists in a thermal superposition at time t of

$$\varphi(x,t) \propto \sum_{n=0}^{\infty} e^{-\frac{(n+\frac{1}{2})\hbar\omega}{2k_B T}} e^{-i\left(n+\frac{1}{2}\right)\omega t}\phi_n$$

where ϕ_n are the energy eigenstates of the system. The root mean square displacement of the oscillator can be numerically calculated by truncating at a given maximum energy level. This value tends towards $\langle x^2\rangle^{\frac{1}{2}} = 24\ \mathrm{pm}$.

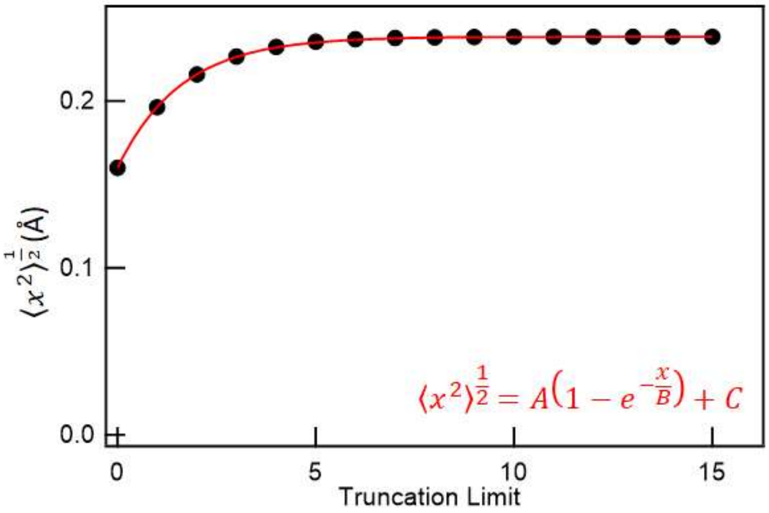

Supplementary Figure 14 | Thermal fluctuations along d_{NAu}. Root-mean-square displacement of adatom along the d_{NAu} coordinate at thermal energy 25meV in a harmonic energy potential approximation with increasing highest-considered energy level.

Around the energy minima, the vibrational energy of the C≡N bond varies locally by 166.4 $cm^{-1}Å^{-1}$ with adatom position, giving an expected thermal variation of approximately 40 cm^{-1}. Given the harmonic approximation and coordinate restriction made here, it is likely that thermal fluctuations drive the observed spectral fluctuations.

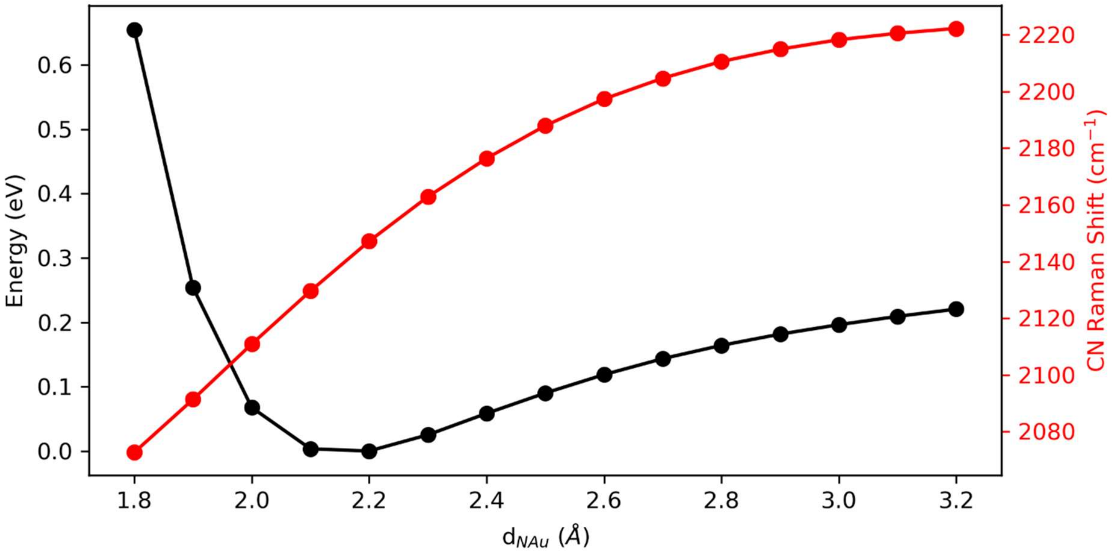

Supplementary Figure 15 | Adatom d_{NAu} energy and CN curve. Relative system energy with varying d_{NAu} with fixed $\theta = 41°, \phi = 90°$ passing through the energy minima. The DFT vibrational energy of the CN bond is also shown along the same curve (scaled by 0.953 to match experiment).

Considering instead motion in ϕ through the minimum energy configuration ($d_{\mathrm{NAu}} = 2.2\text{Å}, \theta = 41°$), the potential landscape can be approximated harmonically for small angle variations from the minima as

$$E = \frac{E_0 \phi^2}{2} \quad , \quad E_0 = 0.14 \text{ eV}$$

giving a harmonic energy level spacing of

$$\hbar\omega = \hbar \sqrt{\frac{E_0}{m\, {d_{\mathrm{NAu}}}^2}} = 2.8 \text{ meV} \approx 3 \text{ meV}.$$

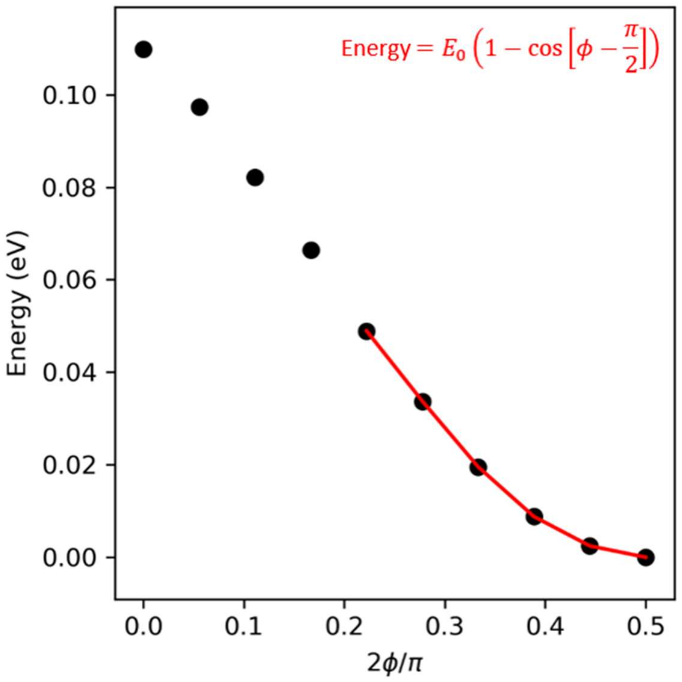

Supplementary Figure 16 | Adatom ϕ energy curve. Relative system energy while varying ϕ with fixed $\theta = 41°$, $d_{\mathrm{NAu}} = 2.2$Å such that curve crosses global energy minima.

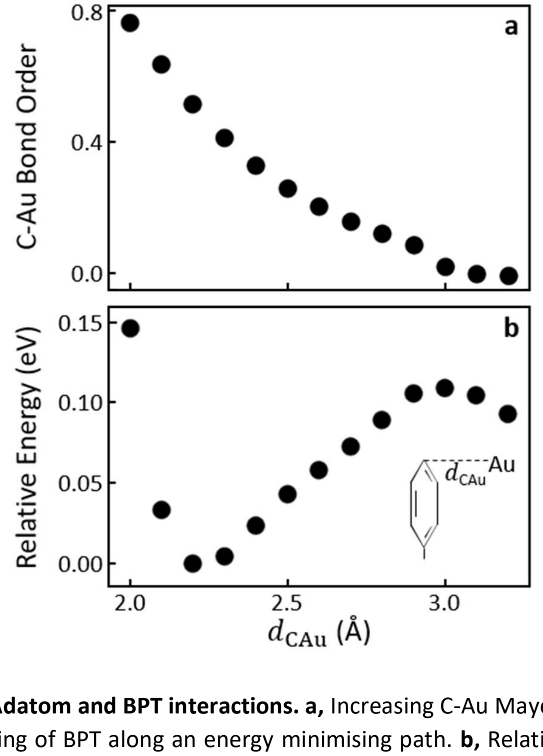

Supplementary Figure 17 | Adatom and BPT interactions. a, Increasing C-Au Mayer bond order as the adatom approaches the top phenyl ring of BPT along an energy minimising path. **b,** Relative DFT system energy along this same path.

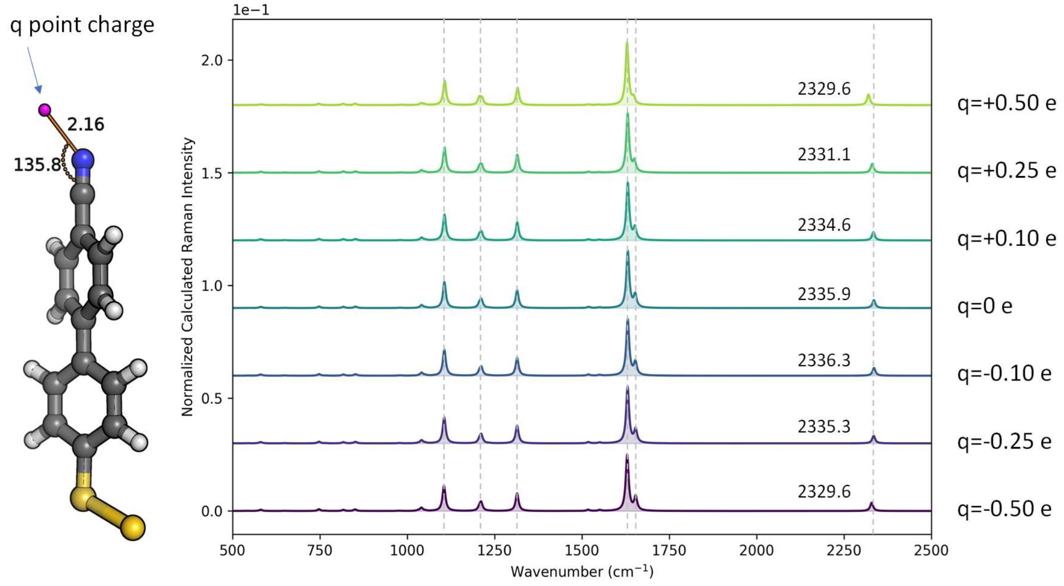

Supplementary Figure 18 | **Effect of a nearby point charge.** Raman spectra calculated for NC-BPT-Au with point charges positioned near the CN group. The position is constrained at the labelled distance and angle. The charge of the point is displayed on the right-hand side next to the corresponding Raman spectrum.

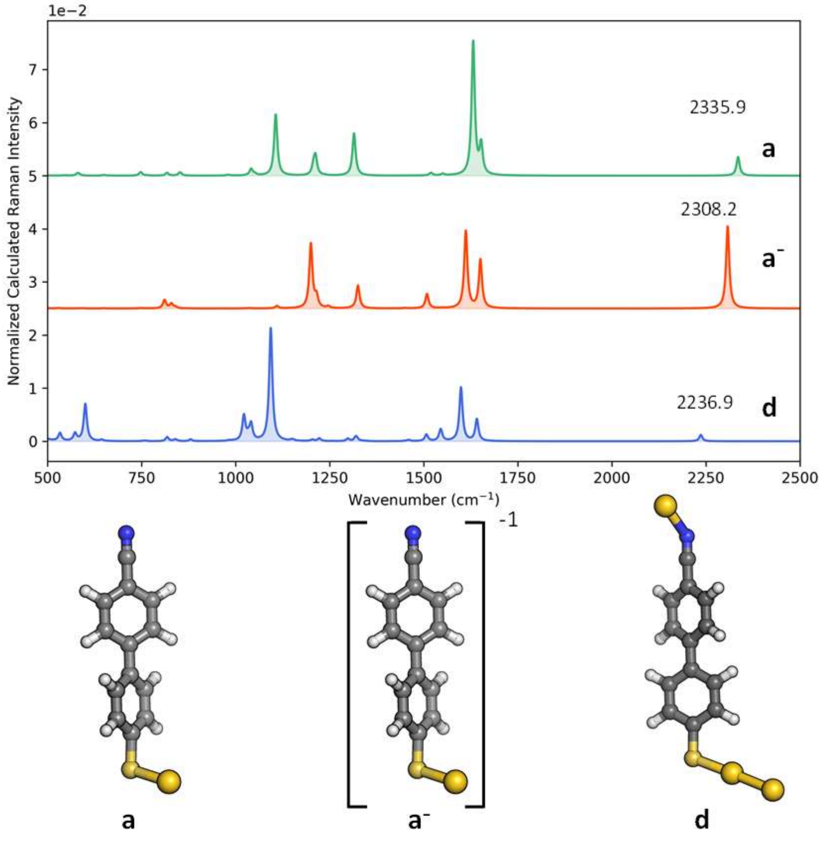

Supplementary Figure 19 | **Effect of molecular charging.** Raman spectra calculated for NC-BPT-Au (**a**), negatively charged NC-BPT-Au (**a**$^-$), and adatom-complexed NC-BPT-Au (**d**).

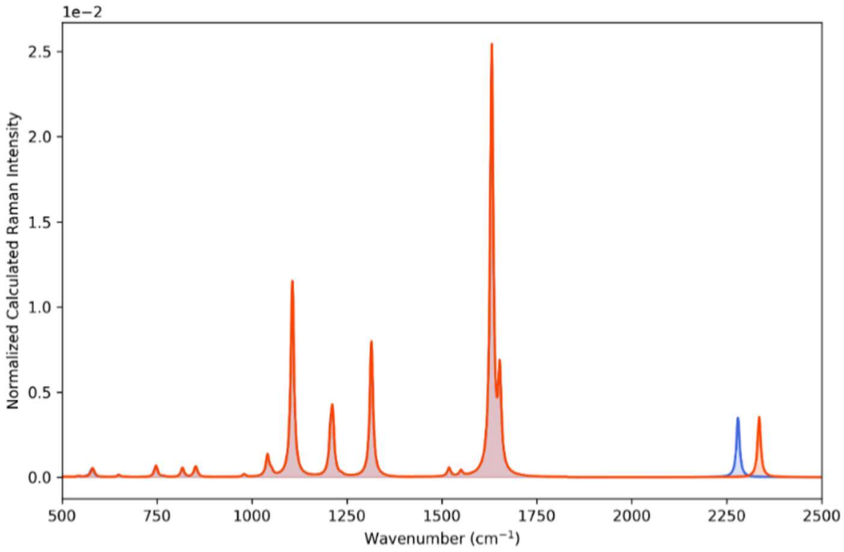

Supplementary Figure 20 | Effect of isotopic substitution. Raman spectrum of NC-BPT-Au for the most abundant isotopes (orange) and with ^{13}C substitution for CN (blue).

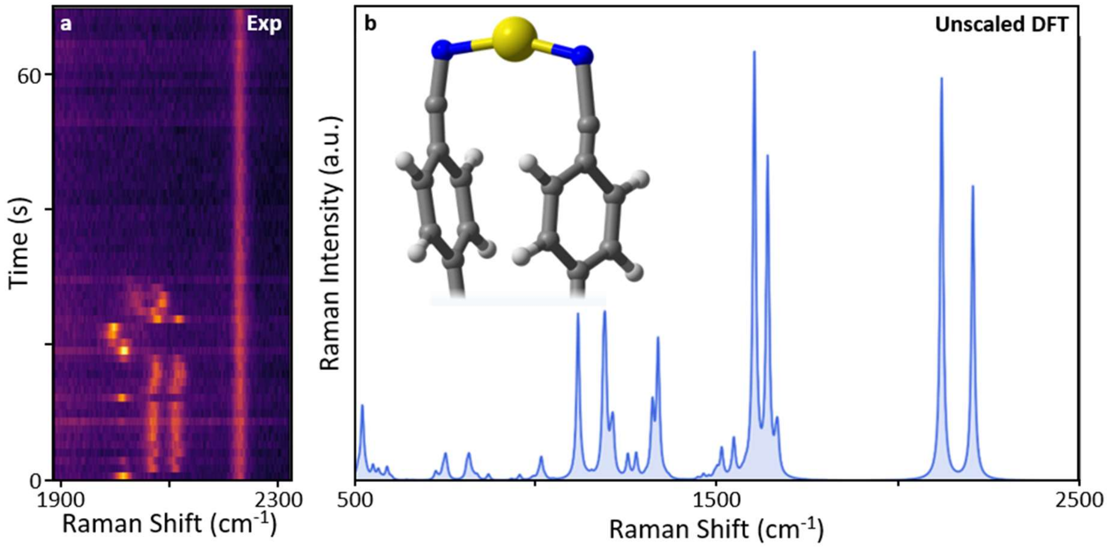

Supplementary Figure 21 | Example of picocavity line splitting. a, Experimental picocavity spectra taken with 1s integration times showing discrete switching between single and spilt vibrational line states. This splitting has multiple possible origins, including sub-integration time switching, the AC Stark effect or **b,** from the vibrational modes of two molecules coupling through mutual interaction with a single adatom.

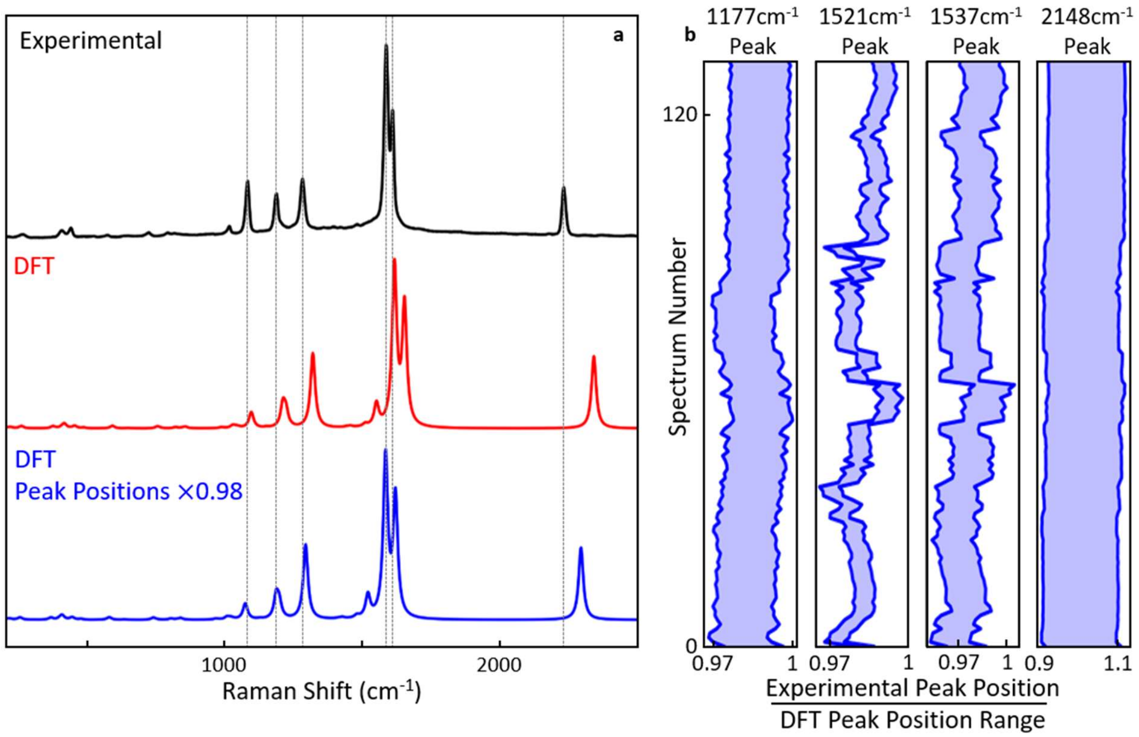

Supplementary Figure 22 | Scaling DFT to Experiment. a, Nanocavity vibrational lines show energy shifts between theory and experiment, both from intrinsic approximations within DFT and due to the application of a simplified model to the complex real environment. Scaling the peak positions by an ad-hoc factor is standard practice that improves agreement, but each peak still contains an error. **b,** When applying individual scaling factors to each picocavity vibrational mode, there are a range of possible factors that would place the experimental value within the range predicted by DFT. For two of the four vibrations however (1521, 1537cm^{-1}), there is no single value valid over all time.

Supplementary Note 4 | Extracting Adatom Positions by Direct Spectrum Comparison

In this section, we show a 'brute force' technique to extract adatom position from exact matches of DFT to picocavity spectra. We demonstrate that this in fact is not tractable due to the current limitations on DFT when using such tightly-confined optical fields. In the following section, we then present an alternative approach.

If there is a very high level of agreement between DFT and experimental peak positions, the relative adatom-molecule position should be extractable by direct comparison of experimental and DFT spectra. It is well known however that the DFT vibrational frequencies must be scaled by an empirical factor to agree with experimental measurements, which varies depending on the level of theory used in the calculation and the character of each vibrational mode.

Here, we describe an attempted method for extracting the most likely conformations of the gold adatom and the NC-BPT- Au-Au group (Au-NC-BPT- Au-Au, ensuring an odd number of gold atoms) by direct comparison of experimental and DFT spectra using the CN vibrational mode and the Ph-vibrational region located at 1400-1700 cm^{-1} in the experimental spectrum. Here, we apply a scaling factor to each of these regions independently. We compare each experimental spectrum in the time

series of picocavity measurements with the set of calculated spectra with the gold adatom placed on a 3D grid around the phenyl (Ph) ring-attached CN group (Supplementary Figure 23).

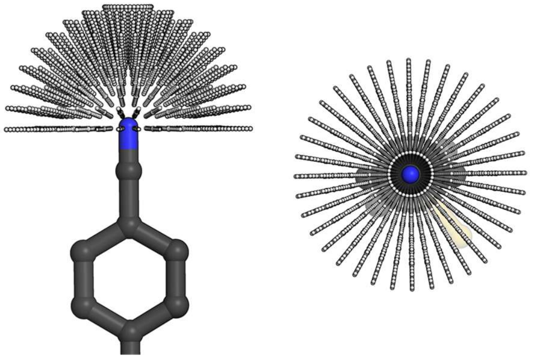

Supplementary Figure 23 | 3D Grid Points for Calculated DFT Spectra. Side and top views of the grid points around the N atom of the NC-BPT molecule. The grid points are mirrored here to all four quadrants around the N atom, assuming an approximate symmetry of the Ph-CN group.

The scaling factor f_{CN}=0.9544 for the CN stretching vibrational peak is set as the peak position ratio between experimental nanocavity measurements and the DFT-calculated vibrational frequency for NC-BPT-Au (Supplementary Figure 24a). A second factor f_{Ph}= 0.9714 is set for the Ph-vibrational region using the average results for the two intensive peaks at 1538.5 cm^{-1} and 1606.8 cm^{-1} (Supplementary Figure 24b). The resulting fit is shown in Supplementary Figure 25. All experimental peak positions are extracted from the measured spectra by fitting a Gaussian function with fixed full width at half maximum (FWHM = 10 cm^{-1}) on the datapoints. A basic baseline-correction is applied using a 5th-order polynomial.

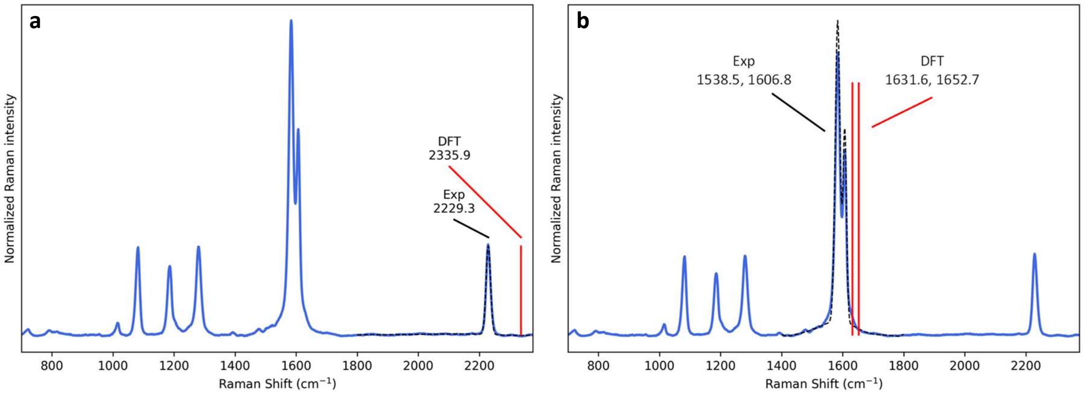

Supplementary Figure 24 | Determining Vibrational Scaling Factors. Comparison of experimental peak positions and DFT calculated nanocavity peak positions (red) to define scaling factors f_{CN} and f_{Ph} for the **a,** CN vibration and **b,** Ph-region (1400cm^{-1} - 1700cm^{-1}). Experimental peak positions are determined using Gaussian fits (black dashed curves).

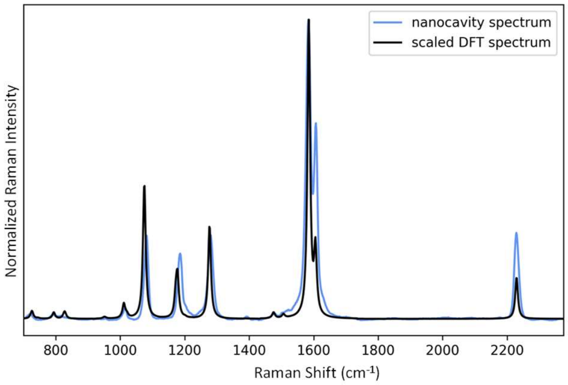

Supplementary Figure 25 | Experimental and Scaled DFT Nanocavity Spectrum. Overlayed normalized Raman spectra from experiment (nanocavity spectrum, blue curve) and DFT calculated Raman intensity spectra (black curve). The DFT calculated spectrum was scaled by f_{Ph} for <1800cm^{-1} region and f_{CN} for >1800cm^{-1}.

For each experimental spectrum, all DFT results are selected with a CN peak position that matches experiment within ≤ 15cm^{-1} accuracy based on the peak width. This reduces the total number of 1350 DFT grid points by ~90%. For the remaining DFT results, the experimental and DFT generated spectra are now directly compared in the Ph-peak region using the spectral angle (α) scoring function:

$$\alpha = \cos^{-1}\left(\frac{\sum_{i=1}^{nb} t_i r_i}{\sqrt{\sum_{i=1}^{nb} t_i^2}\sqrt{\sum_{i=1}^{nb} r_i^2}}\right),$$

where t and r are the two spectral intensity vectors and nb is the number of datapoints in each spectrum. This metric decreases with increasing similarity. Each generated spectrum is formed from the nanocavity spectrum plus Gaussian peaks of fixed FWHM=10cm^{-1} at the positions given by the picocavity calculation. As the altered Raman selection rules caused by the large picocavity optical field gradient are not included in DFT calculations, we expect no agreement between the relative peak intensities seen in DFT and experiment. For each DFT spectrum, the relative Raman intensities are instead set to minimize α. Specific examples are shown for the lowest (3.71°) and highest (15.75°) α values obtained across all experimental spectra considering the best matching configuration from the reduced set of DFT grid points (Supplementary Figure 26). In the resulting spectra the highest picocavity intensities are assigned to the 2nd and 3rd highest frequency peaks in the Ph-region.

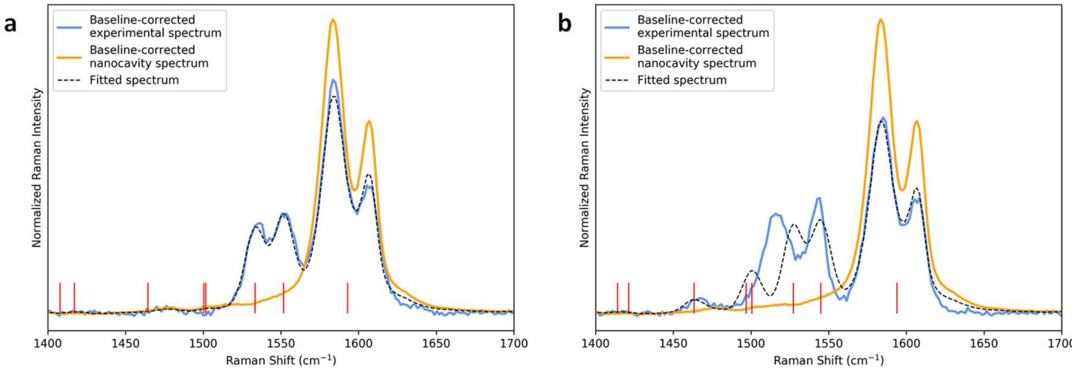

Supplementary Figure 26 | Matching the Ph-Region. Experimental and DFT spectra for picocavity spectra with the **a,** lowest and **b,** highest optimized spectral angle values obtained across all experimental spectra. The red vertical lines represent the positions of the DFT calculated vibrational frequencies.

In Supplementary Figure 27, we depict the spectral angle scores obtained for the prefiltered grid points. For a specific coordinate, we show only the best value along the other two coordinates. These plots demonstrate that this type of fitting tends to give better spectral angle scores for shorter N-Au distances and smaller deviation from the CN-axis at the edge of the available parameter space. If applying a particular scaling factor tended to systematically under- or over-estimate the frequency of a significant vibrational mode such that the experimental value is removed from the possible range provided by the theory, this behavior of returning the adatom position that maximally perturbs the system might be expected. While the experimental spectra vary dynamically over time, this method results in inverted adatom positions that are largely static. While the described methodology is fundamentally sound, these results suggest that attempting to directly match experimental and theoretical spectra requires an even greater level of precise agreement between them. The neglected complexities of the physical system – such as intermolecular interactions and interactions between the molecule and bulk gold – will have to be included before a direct comparison method such as that described here can return a reliable inverted trajectory.

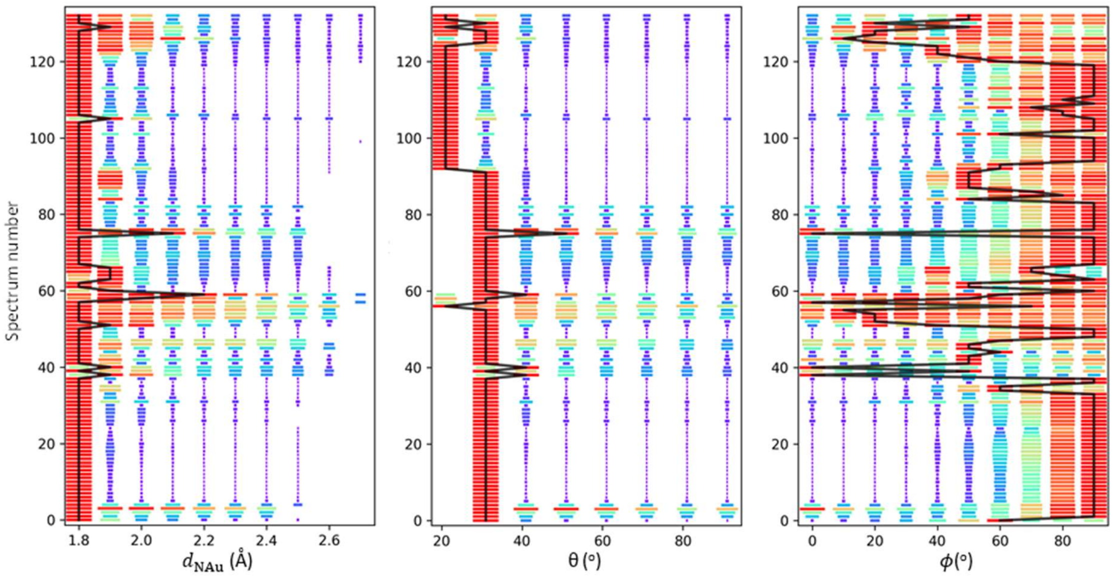

Supplementary Figure 27 | Optimized spectral angle values collected for the reduced grid points. The lowest spectral angle values for **a,** d_{NAu}, **b,** θ and **c,** ϕ polar coordinates of the pre-filtered grid points. Only the lowest spectral angle value is indicated for each coordinate from within all possible values of the other two polar coordinates. Size and coloring refer to the relative score obtained: longer and red-colored bars represent lower spectral angle, and shorter and blue-colored bars represent higher spectral angle. The black continuous lines connect the points with the lowest corresponding spectral angle of each frame of the spectral trajectory. This trajectory almost completely stays at the edge of the parameter space, indicating its unreliability.

Supplementary Note 5| Optimising Adatom Trajectory

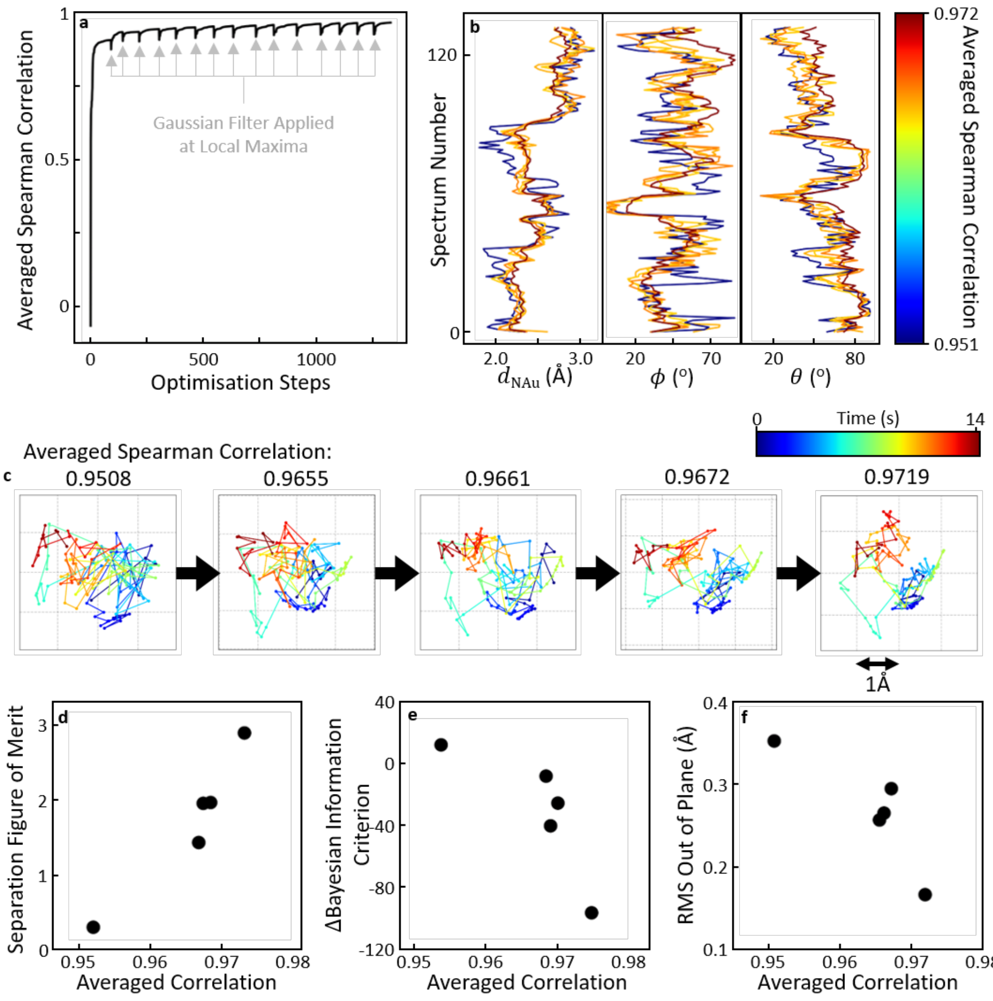

Supplementary Figure 28 | Adatom Trajectory Optimisation. a, Example of the optimisation score of an adatom trajectory with optimisation steps. A Gaussian filter (std=1) is applied with respect to time at each local maximum, allowing the system to escape the local trap and optimise further. **b,** Resulting trajectories shown from optimising with 5 random starting positions. These vary in final score but are of similar form indicating that a global solution exists. **c,** Each trajectory projected into a 2D plane, showing the solution converging on a two cluster configuration with increasing Spearman correlation. Applying a two-part Gaussian Mixture Model to these points in the plane, we can define **d,** a Figure of Merit by the ratio of the cluster separation to the sum of the cluster standard deviations along the line between the cluster centres and **e,** a difference in Bayesian Information Criterion between a two and one cluster Gaussian Mixture Model. A negative value indicates that the system is better described by two cluster sites. **f,** With increasing averaged correlation, the trajectory becomes confined to a 2D plane

In order to extract adatom dynamics information without scaling the DFT to match experimental peak positions precisely, an adatom trajectory through the parameter space is found that maximises the averaged Spearman correlations between the 4 resolvable experimental picocavity lines and their DFT counterparts. This uses the spectral dynamics of the lines, rather than their absolute positions.

Initially, the trajectory is defined by a random position in the parameter space at each point in time. This is then optimised through maximising the averaged Spearman correlation by a random walk process followed by gradient ascent to a local maximum. As each time point was initialised independently, this locally optimised solution contains many sudden position changes that provide a barrier to further optimisation. By applying a Gaussian filter in time (standard deviation = 1 time step), the total score of the trajectory slightly drops (Supplementary Figure 28a). By re-optimising from this position, the system may then be able to optimise to an improved local maximum. If not, the system returns to the previous local maximum. This is repeated until the system cannot, after 2 attempts, improve upon the local maximum found. An illustrative example of the system score while optimising is shown in Supplementary Figure 28a. It should be noted that the averaged Spearman correlation here is a highly non-linear score function - improvements to the trajectory needed to increase the score rapidly become more difficult when approaching the maximum score of 1. This process is run from 5 independent random starting trajectories to ensure that the results were of a similar form and that the solution is therefore unique (Supplementary Figure 28b). The trajectory with the highest score is selected as the optimised solution. The effect on constraining the optimisation was briefly explored by fixing values of ϕ. This resulted in similar trajectories for the remaining parameters as in the unconstrained case, although now varying over larger ranges to compensate for the fixed angle. The resulting averaged correlation drops upon application of these constraints.

Each independently optimised trajectory represents closely spaced local maxima in the parameter space. It is impossible to know if the optimal solution found here represents the global maximum or is simply very close to it. This uncertainty drops with the number of independently optimised trajectories. A full explicit parameter sweep to find the global maximum is infeasible due to the high dimensionality of the parameter space (3 x number of spectra). Each optimised trajectory can be projected into a plane that minimises the total deviation perpendicular to the plane. Ordering these with increasing averaged Spearman correlation, the two clusters become increasingly well defined (Supplementary Figure 28c). This suggests that the two cluster configuration in the plane is the convergent solution with increasing averaged correlation. This convergence can be quantified by separating the points in the plane into two clusters using a 2-part Gaussian Mixture Model. Comparing the inter-cluster separation to the cluster widths along the line between them, the clusters become increasingly resolved with increasing averaged correlation (Supplementary Figure 28d). The Bayesian Information Criterion (BIC) is a numerical metric for comparing how well a set of data is described by a model which decreases with model fitness. Comparing 2-part Gaussian Mixture Models to 1-part Gaussian Mixture Models, the difference in BIC grows strongly with an increase in averaged Spearman correlation (Supplementary Figure 28e).

To probe the robustness of the two clusters, artificial noise was drawn from a normal distribution of standard deviation 0.5 cm^{-1} and applied to each experimental vibrational line (Supplementary Figure 29). The resulting trajectories are less neat and have lower averaged Spearman correlations, as expected. However, the two cluster feature persists.

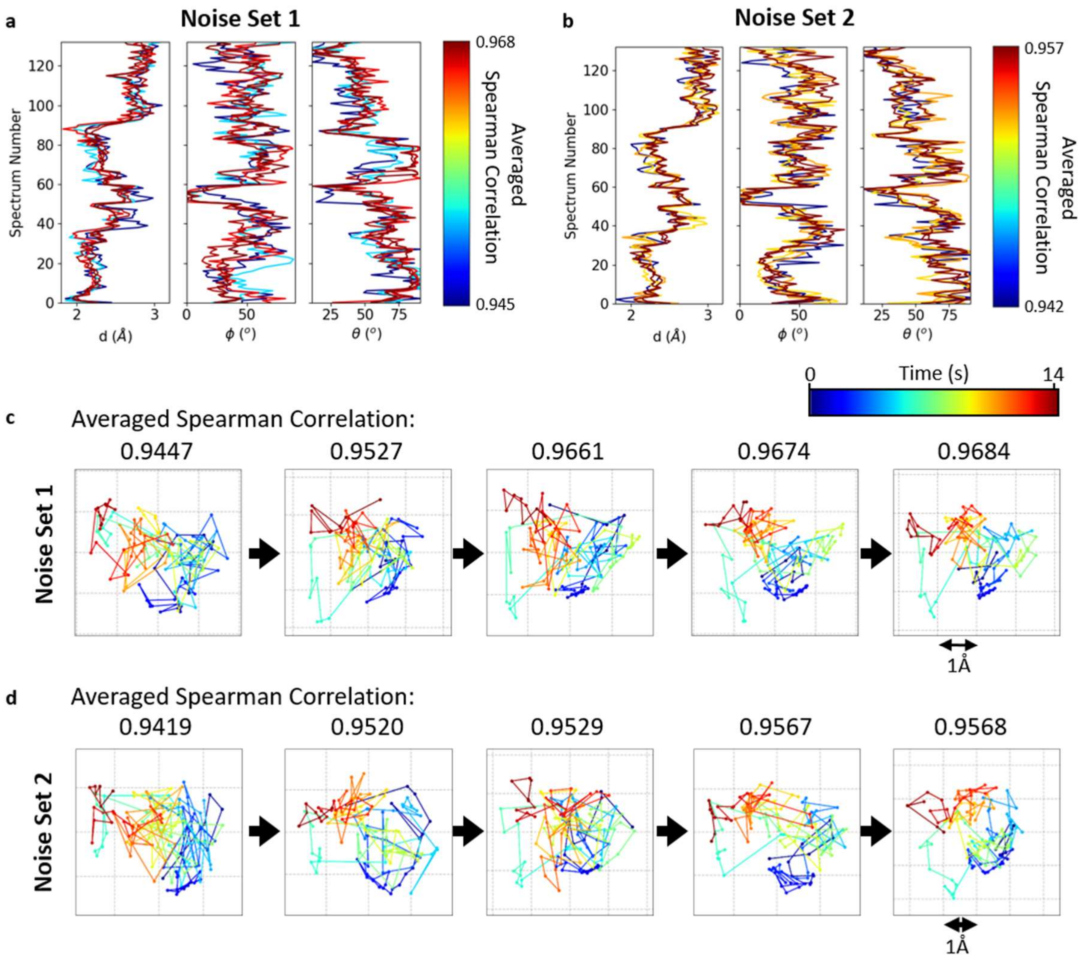

Supplementary Figure 29 | Applying artificial noise. a,b, Optimised trajectories after artificial noise drawn from normal distributions of standard deviation 0.5 cm^{-1} is added to the experimental peak frequencies. The trajectories are noisier and have lower averaged Spearman correlations, but are similar to the trajectories without noise. **c,d,** The trajectories projected into planes. These 2D trajectories are noisier, but the two cluster feature persists.

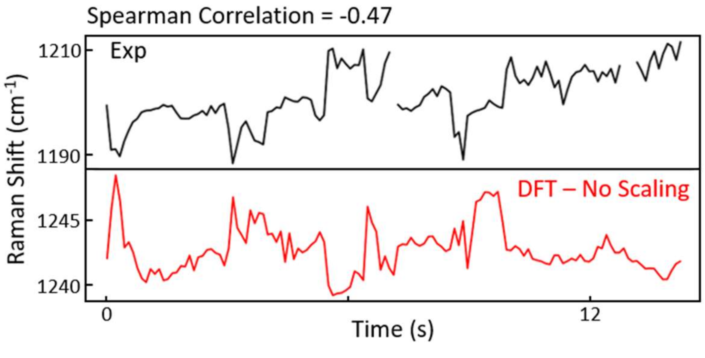

Supplementary Figure 30 | Validation Peak. An experimental peak, not resolvable for all time, and not used in the trajectory optimisation, is examined. Its DFT trajectory predicts dynamics for the peak that are (well) anti-correlated to what is observed. The reasonable absolute correlation indicates that structural dynamics is being reproduced, but the inverted direction further points to missing interactions or parameters such as intermolecular interactions.

Peak Assignment

Here, we use 4 picocavity peaks at average experimental positions 1177 cm^{-1}, 1521 cm^{-1}, 1537 cm^{-1} and 2148 cm^{-1}. As there are no other vibrational modes in the ∼2000 cm^{-1} region, the 2148 cm^{-1} peak can be unambiguously assigned to the CN vibrational mode. In the picocavity spectra, a nanocavity peak at 1184 cm^{-1} becomes 3 visible modes, the lower of which is the 1177 cm^{-1} peak. In the nanocavity DFT spectrum, a reasonable scaling factor of ∼0.971 aligns a close triplet of peaks to this experimental region (Supplementary Figure 31a). The central bright mode is not strongly perturbed by the adatom. This is flanked by two dark modes that are more strongly perturbed by the adatom. Therefore, the 1177 cm^{-1} mode is assigned to the lowest energy DFT mode of this triplet. The higher frequency mode, which is not resolvable for all time, is the *validation peak* assigned to the higher energy mode of this triplet.

The vibrations at 1521 cm^{-1} and 1537 cm^{-1} exist in a region of the spectrum containing vibrations of the phenyl rings. Zero-indexing the modes in the nanocavity DFT from lowest to highest energy, there are 8 phenyl ring vibrations indexed 55-62 (inclusive) consisting of 4 sets of symmetric/anti-symmetric pairs. Vibrations 61/62 represent modes bright in the nanocavity spectrum. Some modes can be immediately excluded as they require the DFT vibrations to be scaled up instead of down to match the same range as experiment. This would disagree with all other visible nanocavity modes which are overestimated in frequency by the DFT. Still, some ambiguity to peak assignment remains. In the trajectory presented in the manuscript, vibrations 59/60 are assigned to these experimental peaks. These require an approximate scaling of 0.97 to 0.98 to overlap with the region seen in experiment and represent the next pair of modes down in energy from those bright in the nanocavity spectrum. Here, we briefly explore alternative peak assignments to vibrations 58/60, 59/60, 60/61 and 61/62 (Supplementary Figure 31). The resulting trajectories are similar to those found with our assignment, suggesting some redundancy in the information contained in these phenyl ring vibrations. However, the resulting averaged Spearman correlations are worse and are in some cases only as high as the worst Spearman correlation found with our assignment. Each of these alternative assignments predict the validation peak as anti-correlated to what is observed experimentally.

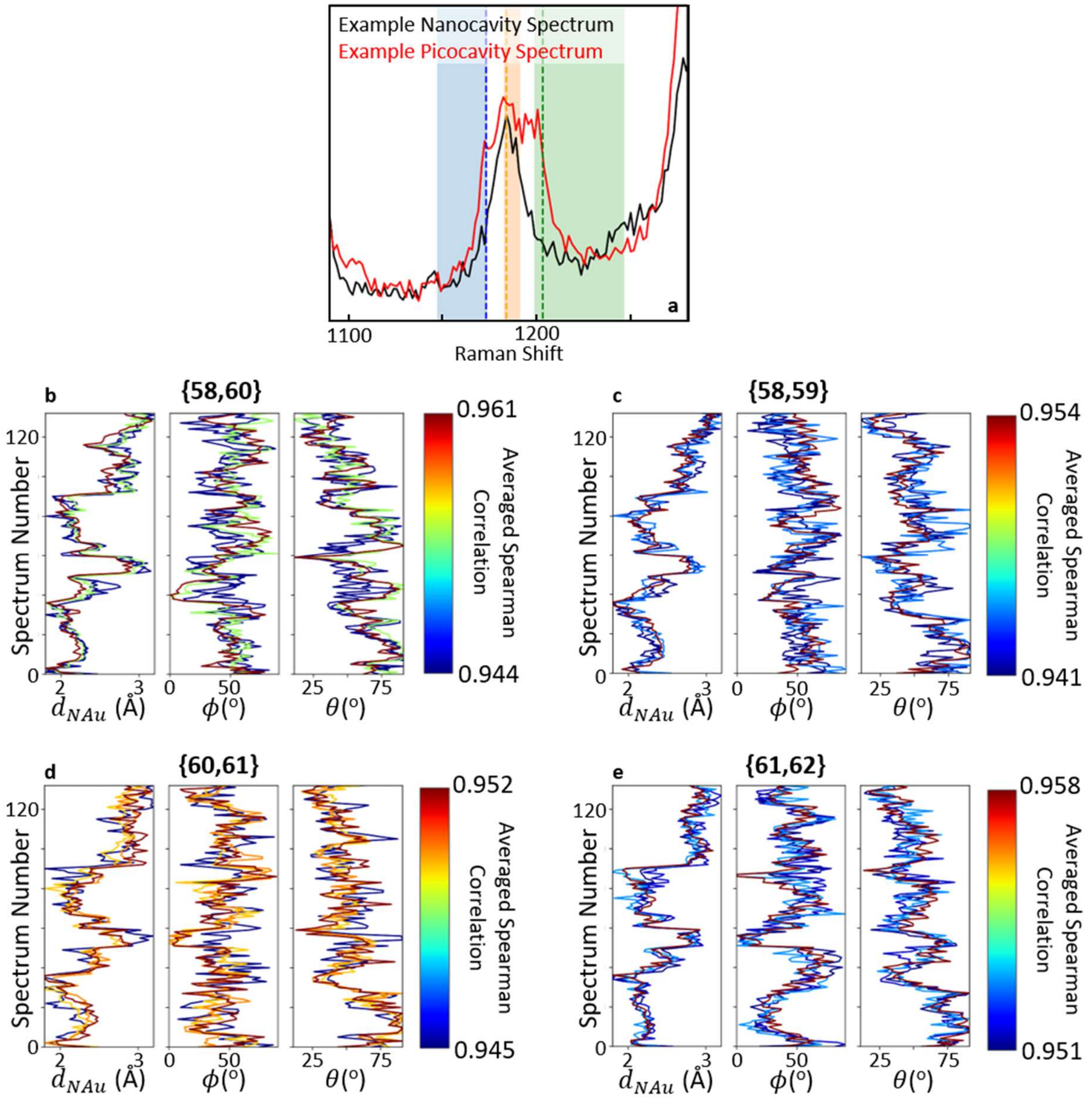

Supplementary Figure 31 | DFT Mode Assignments. a, Experimentally, a single nanocavity peak near 1200 cm^{-1} is replaced with 3 peaks in the picocavity spectra. This matches with a triplet of lines in the DFT calculations where only the central peak is bright in the nanocavity spectrum. Dashed lines indicate calculated nanocavity line positions when the DFT is scaled by 0.971. Shaded regions indicate the range of perturbed peak positions available within the DFT dataset. Trajectories can be generated where the phenyl ring modes at 1521 cm^{-1} and 1537 cm^{-1} are assigned to different DFT modes. Zero-indexing the modes from lowest to highest nanocavity energy, we extract trajectories when assigning these experimental lines to modes **b,** 58 and 60, **c,** 58 and 59, **d,** 60 and 61 and **e,** 61 and 62. These all provide similar trajectories to the utilised assignment of 59 and 60, but with lower averaged Spearman correlations.

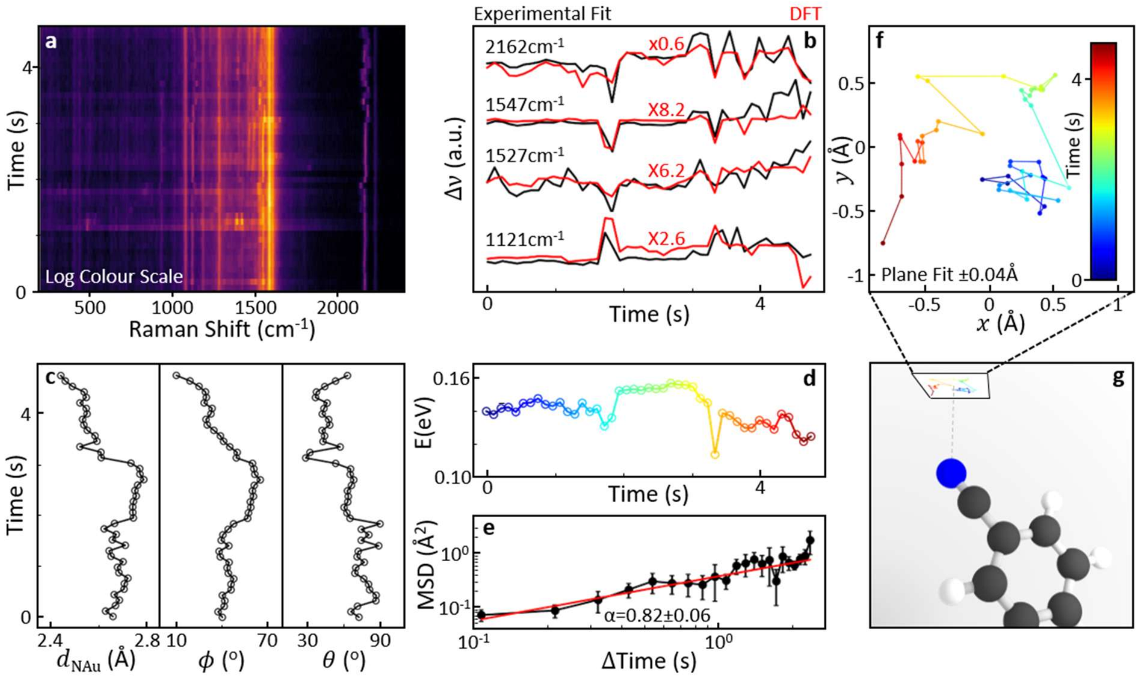

Supplementary Figure 32 | Additional example adatom track from experimental spectra. a, Experimental picocavity spectra taken with 300µW 633nm laser power with 100ms integration time (log scale). Time shown since detected picocavity formation. **b,** Extracted picocavity peak positions, overlaid with corresponding peak positions from an optimised adatom trajectory in DFT. These are plotted on individual scales to emphasise the shape of the peak trajectories. **c,** Extracted adatom coordinates for this trajectory. **d,** DFT system energy along adatom trajectory**.** Colours refer to time colour bar. **e,** Mean Square Displacement (MSD) of adatom with varying time windows. Error bars indicate standard error. **f,** Adatom trajectory projected onto a plane and **g,** plane shown relative to molecule in 3D. Colours refer to time colour bar.

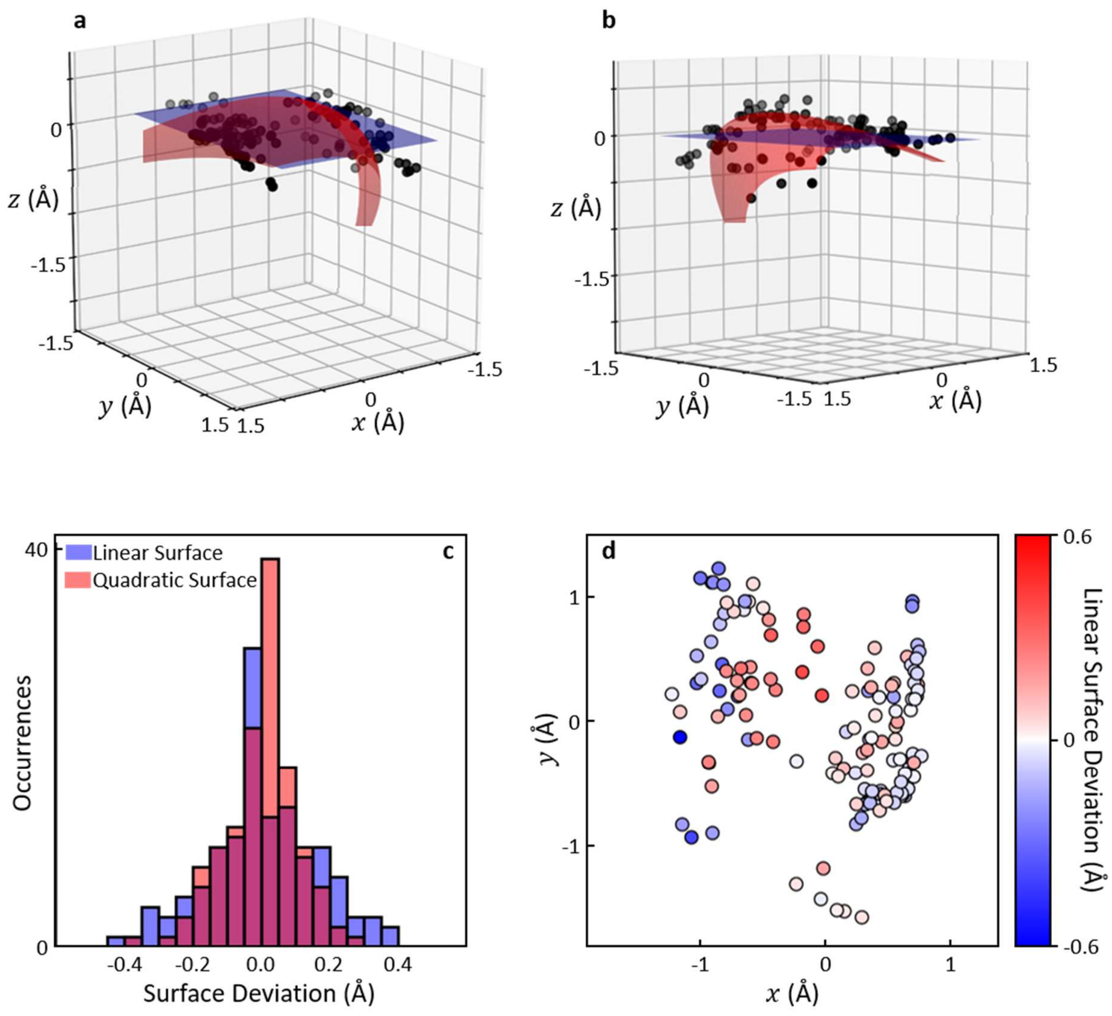

Supplementary Figure 33 | Plane of the extracted adatom trajectory. a,b, Extracted adatom positions, alongside optimisations on linear (blue) and quadratic (red) surfaces. The optimised quadratic plane shows curvature of -1.25Å^{-1} and -0.15Å^{-1} in two orthogonal directions. The lower plane of the figures represents the height of the molecular nitrogen at the end of the NC-BPT molecule. **c,** Deviations between adatom positions and optimised surfaces. The linear surface is shown to be a valid projection with an error of ±0.17Å. The quadratic projection reduces this to ±0.11Å at the expense of complexity. **d,** Signed deviation of linear surface projection with projected position

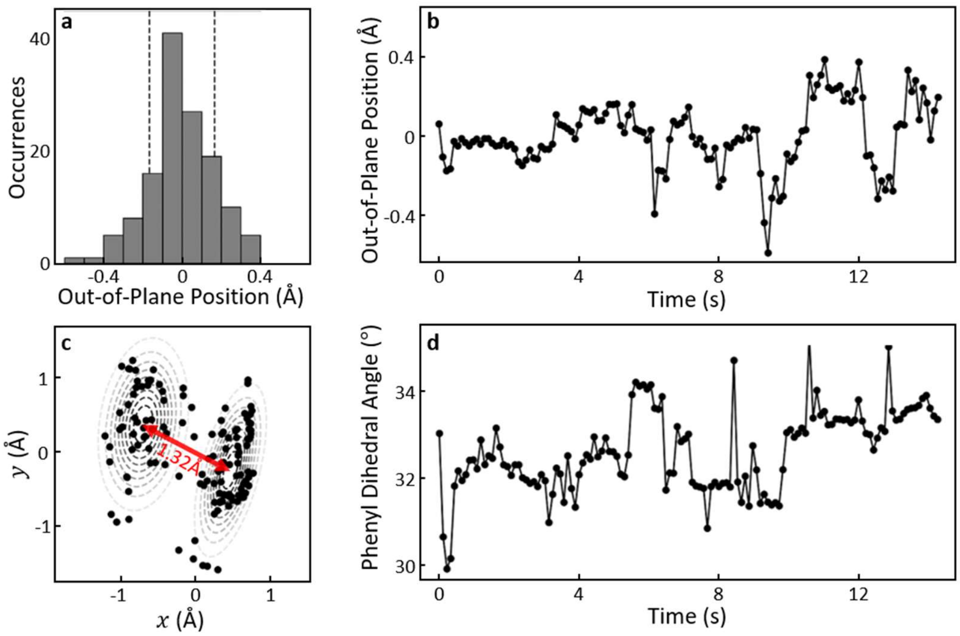

Supplementary Figure 34 | Properties of the extracted adatom trajectory. a, Histogram of adatom deviation away from an optimised plane and **b,** this deviation over time. **c,** Adatom positions within the plane, clustered into two groups using a two-part Gaussian Mixture model. This model is used to define how the atoms of a {111} surface overlay on this plane. **d**, The dihedral angle between the NC-BPT phenyl rings over time, as calculated by the DFT.

Supplementary Note 6 | Sensitivity of Results on DFT Parameters

The DFT calculations in this work with varying adatom-molecule position are calculated using the Def2-TZVP basis set, B3LYP hybrid density functional and D3 dispersion correction (doi: 10.1063/1.3382344) with Becke-Johnson damping (doi: 10.1002/jcc.21759). It is shown that there is an expected discrepancy between the exact vibrational energies predicted by the DFT model and experiment. This is both due to modelling a simplified system and the internal approximations that each DFT hyper-parameter represents. Extracting an adatom trajectory from SERS using the Spearman correlation between experimental and calculated SERS mode dynamics is designed to be less sensitive to the exact vibrational energies predicted. Here, this is tested by comparing the DFT response while varying these hyper-parameters. For key changes to the basis set and exchange functions used in the DFT calculations, the full parameter space of relative adatom-molecule position is recalculated and the adatom trajectory is extracted.

To compare the influence of the basis set, the Raman spectra for both the Au-BPT-CN and Au2-BPT-CN-Au system are recalculated in the energetically minimising geometries using the Def2-SVP and Def2-QZVP basis sets. The Au2-BPT-CN-Au spectra are compared over the spectral range 1200-2350 cm^{-1} (relevant for adatom trajectory extraction) in comparison to Def2-TZVP used in the manuscript. In each case, the calculated spectra are scaled in vibrational energy by comparing the experimental nanocavity spectrum to the Au-BPT-CN vibrational energies. This utilises two high intensity peaks in the experimental spectrum - assigned to ring vibrational modes - at 1583.5 cm^{-1} and 1606.8 cm^{-1} to define a scaling factor f_{Ph} for the region $\leq$1800 cm^{-1}. The CN vibrational mode defines a scaling factor f_{CN} for >1800 cm^{-1}. The overlay of the three spectra (Supplementary Figure 35) reveals

no significant differences between the Def2-TZVP and the more computationally expensive Def2-QZVP basis sets regarding peak positions and even relative intensities. While there is a small shift in the CN vibrational mode energy, from these calculations we conclude that the computational cost of extracting the adatom trajectory with the larger Def2-QZVP basis set is not justified. Meanwhile, the Def2-SVP peak positions differ significantly especially in the region 1400-1510 cm^{-1}. Therefore, the adatom trajectory is extracted using this basis set as permitted by the lower cost of the calculations at this level of theory.

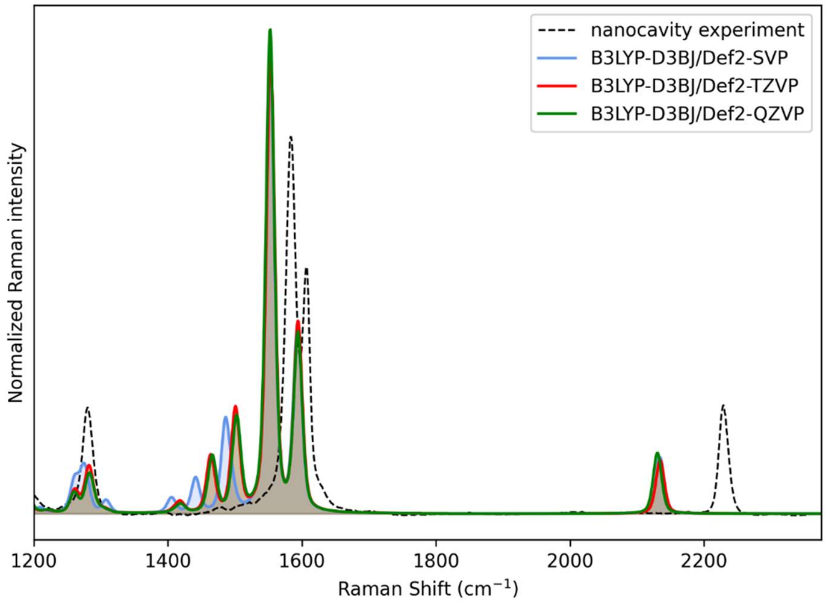

Supplementary Figure 35 | Varying DFT basis set. Calculated Raman spectra for the Au2-BPT-CN-Au system with the Def2-SVP, Def2-TZVP and Def2-QZVP basis sets. Scaling factors are applied separately to the regions ≤1800cm^{-1} (f_{Ph}) and >1800cm^{-1} (f_{CN}) by comparing the experimental nanocavity spectrum (dashed) to the calculated spectra of the Au-BPT-CN system. These are: Def2-SVP: f_{Ph}=0.9616, f_{CN}=0.9444; Def2-TZVP: f_{Ph}=0.9714, f_{CN}=0.9544; Def2-QZVP: f_{Ph}=0.9727, f_{CN}=0.9556.

Using the Def2-TZVP basis set, we also change the DFT functional from B3LYP to the B3PW91-D3BJ (doi: 10.1063/1.464913) hybrid and the M06-L-D3 (doi: 10.1063/1.2370993) meta-GGA functionals. These B3PW91 calculations include the Grimme's D3 dispersion correction with Becke-Johnson damping. Benchmark calculations demonstrate that B3PW91 can surpass the performance of B3LYP in the accuracy of higher (>1000 cm-1) frequencies with larger basis sets (doi: 10.1002/jcc.23073). The M06-L functional has been demonstrated (doi: 10.1039/B810877C, 10.1063/1.4936654) to provide vibrational frequencies with reasonable accuracy in general and for conjugated molecules in particular (doi: 10.1021/acs.jctc.9b01068). These changes lead to more significant shifts in the adatom perturbed vibrational energies (Supplementary Figure 36). The full DFT parameter space was recalculated with each of these functional changes with the range of possible perturbed Raman spectra shown in Supplementary Figure 37.

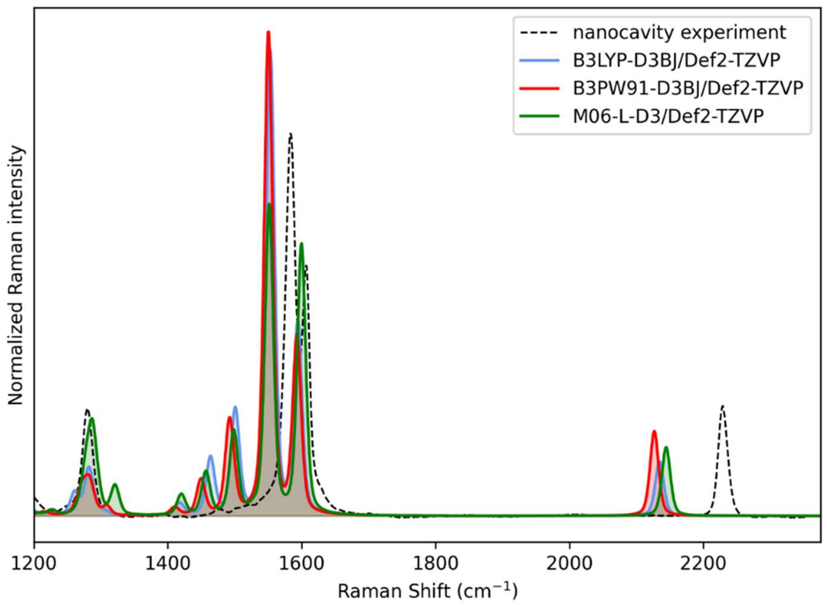

Supplementary Figure 36 | Varying DFT functional. Calculated Raman spectra for the Au2-BPT-CN-Au system with the B3LYP, B3PW91 and M06-L functionals. Scaling factors are applied separately to the regions ≤1800cm^{-1} (f_{Ph}) and >1800cm^{-1} (f_{CN}) by comparing the experimental nanocavity spectrum (dashed) to the calculated spectra of the Au-BPT-CN system. These are: B3LYP-D3BJ: f_{Ph}=0.9714, f_{CN}=0.9544; B3PW91-D3BJ: f_{Ph}=0.9643, f_{CN}=0.9510; M06-L-D3: f_{Ph}=0.9666, f_{CN}=0.9637.

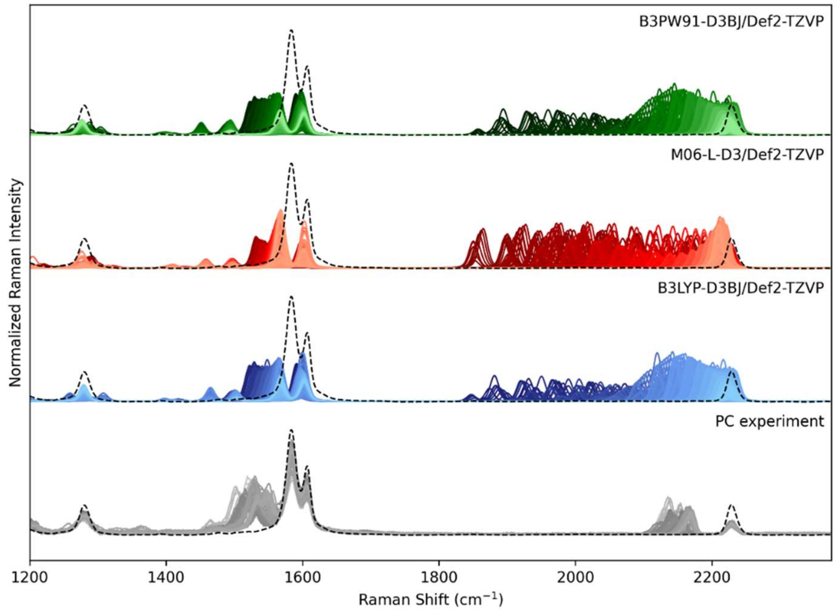

Supplementary Figure 37 | Perturbed Spectra with Varying Functional. Range of modelled Raman spectra (blue, red, green) for the Au2-BPT-CN-Au structure with varying DFT functional and adatom position. Scaling factors are applied separately to the regions ≤1800cm^{-1} (f_{Ph}) and >1800cm^{-1} (f_{CN}) by comparing the experimental nanocavity spectrum (dashed) to the calculated spectra of the Au-BPT-CN system. These are: B3LYP-D3BJ: f_{Ph}=0.9714, f_{CN}=0.9544; B3PW91-D3BJ: f_{Ph}=0.9643, f_{CN}=0.9510; M06-L-D3: f_{Ph}=0.9666, f_{CN}=0.9637. Experimental picocavity spectra are shown in grey.

For each of the DFT parameter changes (basis set to Def2-SVP and functional to B3PW91 and M06-L), sets of five adatom trajectories are independently optimised (Supplementary Figure 38). No significant change in the form of the resulting trajectory is seen, corroborating the hypothesis that this trajectory extraction method is not highly sensitive to these DFT parameters. Again, the ϕ coordinate is observed to be the noisiest as this is the coordinate to which the adatom-molecule interaction is least sensitive.

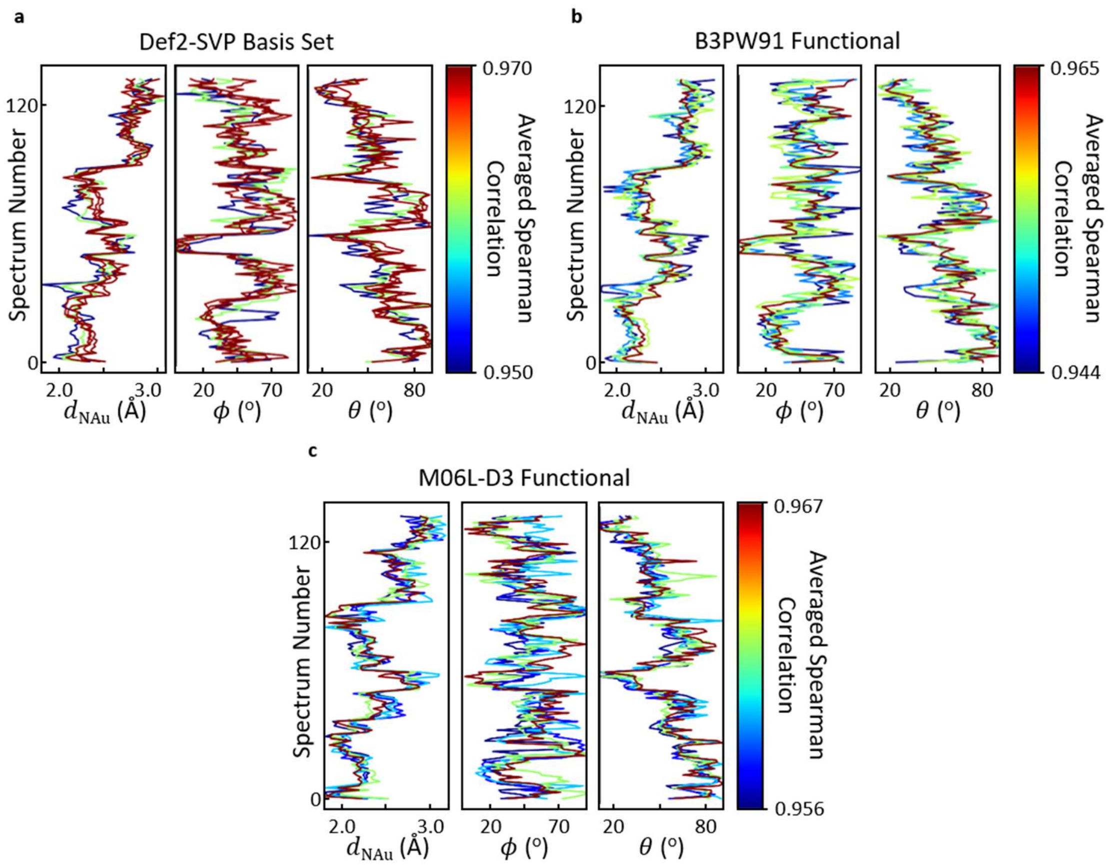

Supplementary Figure 38 | Adatom trajectory with varying DFT parameters. Sets of five optimised adatom trajectories, each initialised randomly, while altering the DFT parameters. This includes changing **a,** the basis set to Def2-SVP and **b,** the functional to B3PW91 or **c,** M06L-D3

Supplementary Note 7 | Gold adatom on a surface

Au adatom positioning on a Au(111) slab was investigated via a 2D potential surface scan (Supplementary Figure 39). At every investigated x-y point, only the coordinate z was optimised, which is the distance between the adatom nucleus and the plane of the gold slab. All atoms in the gold slab were kept frozen in place. The calculations were carried out at B3LYP-D3BJ/Def2-TZVP level of theory. The two interstitial energy barrier heights found (3.3 and 5.0 kcal/mol) are not rotationally symmetric due to the small array of atoms modeled.

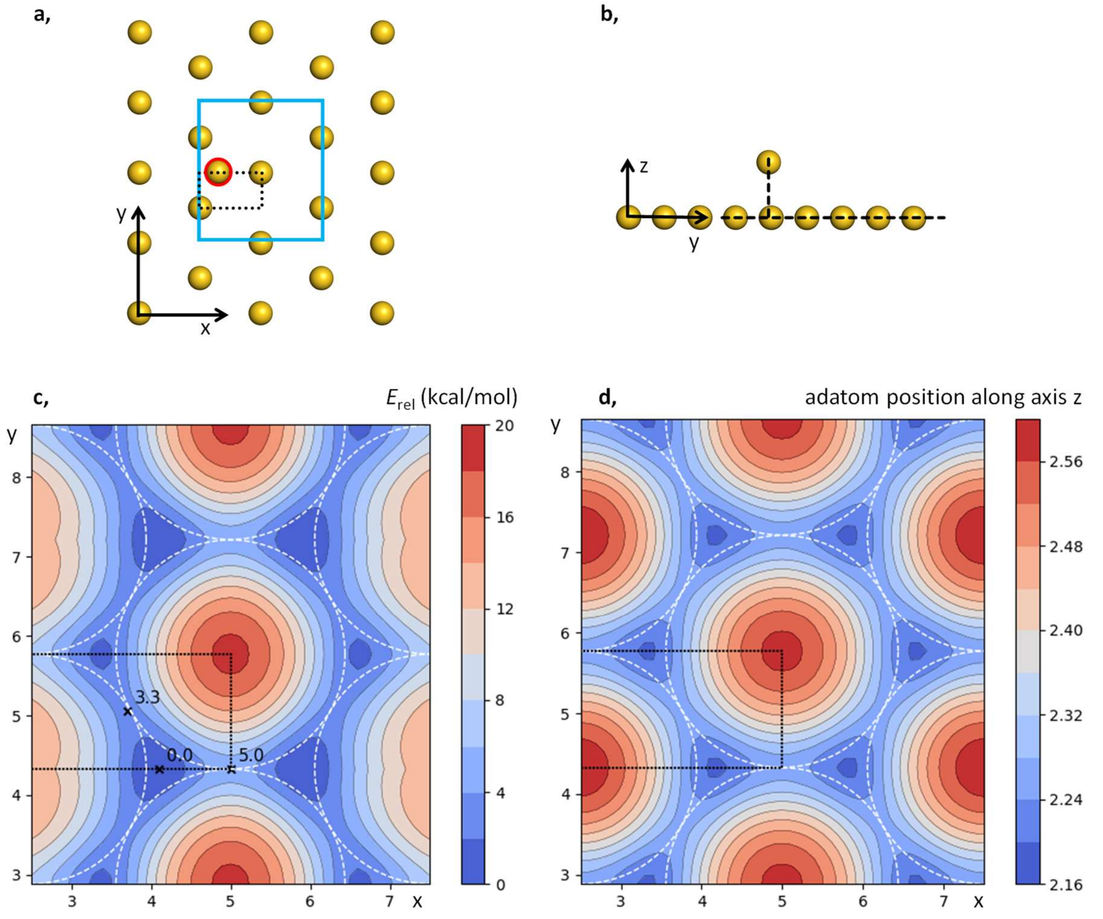

Supplementary Figure 39 | Relative electronic energy and vertical position of Au adatom on Au(111) surface. a, Top view of the calculated system. The black dotted rectangle shows the calculated region, while the blue continuous rectangle shows the area on which the values were extrapolated via reflections. The adatom is highlighted with red outline. **b,** Side view. The vertical position is defined by its z coordinate with respect to the plane of the gold slab. **c,** Electronic energy obtained for different adatom positions. Gold lattice positions marked with white circles, calculated area is bounded by a black rectangle. Energy at the global minimum and the nearby two saddle point as labelled. **d,** Optimized adatom height above the plane.

Supplementary Note 8 | Electronic energy vs. tilting angle for the NC-BPT-Au system

Two-dimensional potential energy surface scan calculations are used to estimate the dependence of electronic energy on the tilting angle of the molecule. In this exploration the bending with respect to the Au-S axis was restricted by using two parameters: 1, the angle between the Au, S and C atoms defining the *level* of tilting, while 2, the dihesdral angle between the Au, S, and the ipso and ortho carbon atom defining the *direction* of tilting. All other degrees of freedom were optimised and the electronic energies were calculated at the B3LYP-D3BJ/Def2-TZVP level of theory. Similar to the adatom tracking, only points in a single quadrant were calculated, and these extrapolated to the remaining three quadrants. In Supplementary Figure 40, the N positions with respect to the S atom are illustrated with translucent spheres, and coloured in accordance with the corresponding electronic energy of the system. This result suggest that the molecule prefers a tilting angle in the range of 60°-80°.

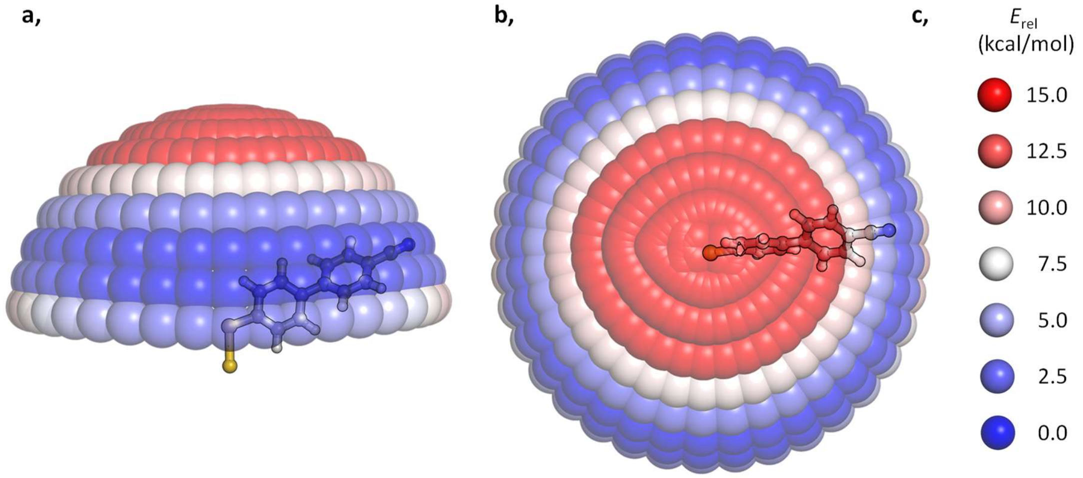

Supplementary Figure 40 | Electronic energy of NC-BPT-Au as a function of the tilting of the molecule. N positions are depicted as translucent spheres. **a,** Side view. **b,** Top view. **c,** Colour scale.

Supplementary Note 9 | Metric for Spectral Dynamics

At a given temperature and incident laser power, a SERS peak from a single molecule varies in energy in time around a central value. We model this by assuming the peak spectral position samples from a normal probability distribution function (PDF) with a standard deviation $\Delta\mu$. While SERS lines are often Lorentzian functions, for mathematical simplicity we here represent the SERS line as a Gaussian peak of standard deviation of σ. Experimentally, a finite integration time Δt is used, which can be modelled by taking $N \propto \Delta t$ samples of the peak position.

If two measurements are taken, the centre positions of the measured peaks will differ by Δx, which follows a half-normal PDF

$$P(\Delta x) = \frac{2}{\sqrt{2\pi W^2}} e^{-\frac{1\Delta x^2}{2W^2}} \qquad W = \sqrt{\frac{2}{N}}\Delta\mu, \qquad \Delta x \geq 0$$

$$\langle \Delta x \rangle = \frac{2}{\sqrt{N\pi}} \Delta\mu \quad .$$

The average measured standard deviation γ follows

$$\gamma^2 = \sigma^2 + \Delta\mu^2 \left(1 - \frac{1}{N}\right) \quad .$$

These measurements can be combined to remove any dependence on integration time

$$\Delta\mu^{\,2} = \frac{\pi}{4} \langle \Delta x \rangle^2 + (\gamma^2 - \sigma^2)$$

which contains the single unknown σ. These relationships are shown numerically in Supplementary Figure 41, which confirms the model derived above.

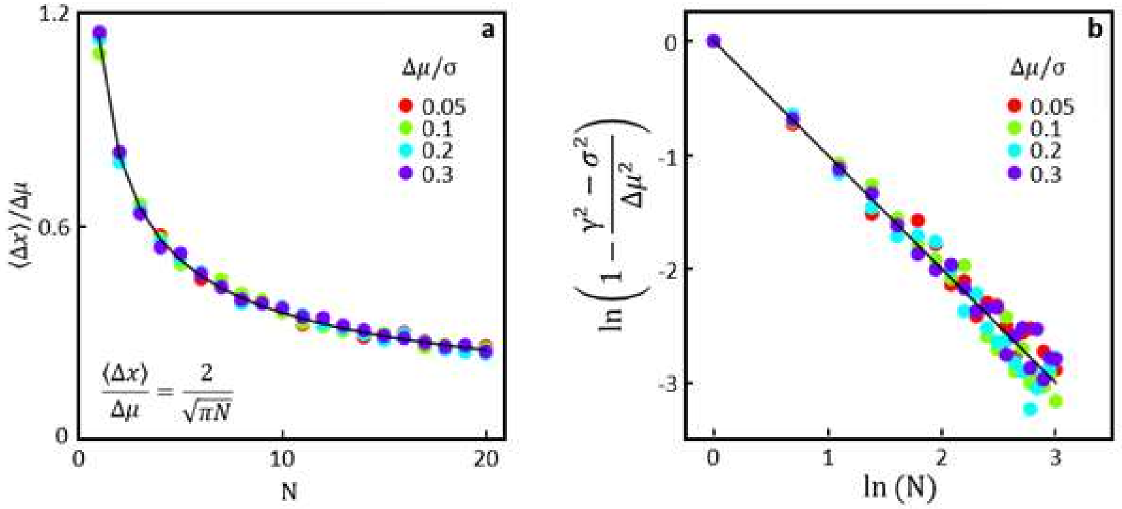

Supplementary Figure 41 | Numerical verification of wandering and peak width. a, Mean position of the sum of N Gaussian peaks of width σ=1, each with random centre position drawn from a normal PDF of width $\Delta\mu$. Each point is the average of 1000 iterations. **b,** the standard deviation γ during this same process. Analytical solutions are shown as black lines.

To extract this spectral fluctuation $\Delta\mu$, we use measurements taken of picocavity lines near 1500cm^{-1} in BPT at 10K (Supplementary Figure 42a). Here, we assume zero thermal broadening (see below). Assuming as typical that $\Delta\mu$ increases exponentially in this regime of temperatures and incident powers, we find the intrinsic $\sigma^2 = 19$ cm^{-2}. Due to the similarity between BPT and NCBPT, this value is used for both molecules. Extracting $\Delta\mu$ for BPT at 300K is done using NPoMs measured at higher laser powers and a short 10ms integration time, averaging over picocavities from a total of 106 NPoMs which were each measured at fixed power.

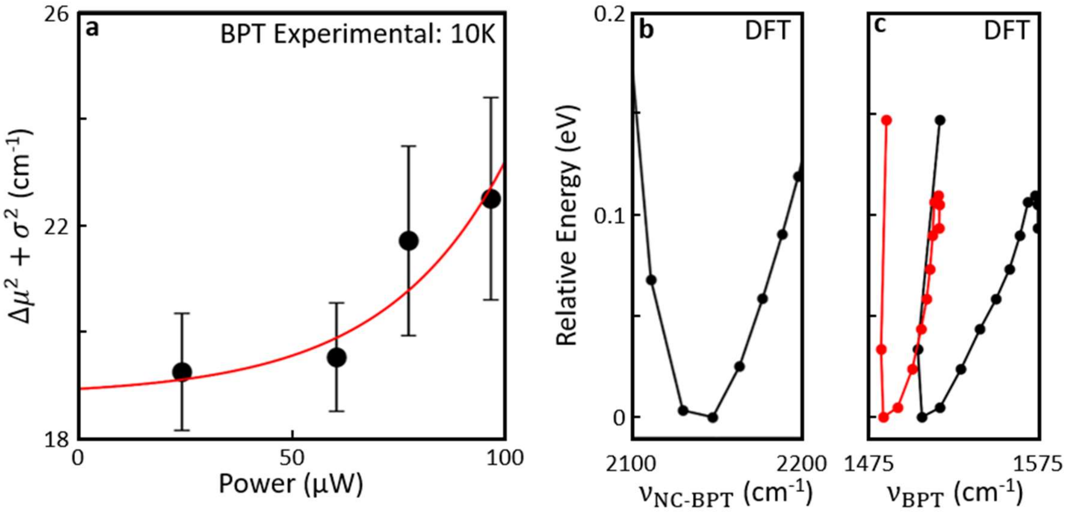

Supplementary Figure 42 | Dynamics of vibrational lines. a, Observed spectral variance $\Delta\mu^2$+σ^2 with increasing laser power for a BPT picocavity at 1513 cm^{-1}, measured at 10K. Assuming no thermal broadening, an exponential fit gives a value of $\sigma^2 = 19$ cm^{-2}. Error bars denote standard error. **b,** Relative energy from DFT of the NCBPT-adatom system vs CN vibrational frequency, with adatom-molecule distance varied (at fixed angular position, frequency scaled by 0.953 to match experiment). **c,** Energy vs vibration frequency for 2 BPT vibrational lines in the spectral region of interest, with the adatom-molecule distance varied along the energy minimising path (in practice angular position is effectively fixed). These frequencies are scaled by 0.97 to match experiment.

With varying adatom-molecule distance, the system energy E and vibrational frequencies ν_i both vary as in Fig.2d,e (main text). Plotting these against each other (Supplementary Figure 42b,c) allows the thermal contributions to be read out. Estimating thermal occupancy $P(E) = A\exp\{-E(\nu)/k_BT\}$, where A is a normalisation factor, we estimate $\Delta\mu$ by the change in frequency around the energy minima that give $P(E) = A/\sqrt{e}$. Around the global energy minimum, small changes in CN vibrational frequency induced by a change in adatom-molecule distance require 2.0 meV/cm^{-1}. This can then directly provide the estimate $\Delta\mu_{\mathrm{NC-B}}$ ~6.3cm^{-1} in good agreement with experiment. The two BPT vibrational lines that lie in the experimental spectral region require 1.4 meV/cm^{-1} and 1.1 meV/cm^{-1}, providing estimates $\Delta\mu_{\mathrm{BPT}}$~8.9 cm^{-1}, 12 cm^{-1}. These overestimate the experimental value by a factor of 3 to 4. This is further evidence that the role of intermolecular interactions and other higher level effects, not captured in the theory, have substantial impact on phenyl ring mode dynamics.
