# Supplementary material for: Resolving sub-angstrom ambient motion through reconstruction from vibrational spectra
Source: Nat Commun. 2021 Nov 19;12:6759. doi: 10.1038/s41467-021-26898-1 (PMC8604935; doi:10.1038/s41467-021-26898-1)
Supplement: Supplementary file 2 — Description of Additional Supplementary Files [file 41467_2021_26898_MOESM2_ESM.docx]

**Description of Additional Supplementary Files**

**File Name:** Supplementary Movie 1

**Description:** The relative adatommolecule trajectory shown in a 2D projection in Figure 3 displayed in 3D in real time.
